# Distinct Biochemical Pools of Golgi Phosphoprotein 3 in the Human Breast Cancer Cell Lines MCF7 and MDA-MB-231

**DOI:** 10.1371/journal.pone.0154719

**Published:** 2016-04-28

**Authors:** María J. Tenorio, Breyan H. Ross, Charlotte Luchsinger, Andrés Rivera-Dictter, Cecilia Arriagada, Diego Acuña, Marcelo Aguilar, Viviana Cavieres, Patricia V. Burgos, Pamela Ehrenfeld, Gonzalo A. Mardones

**Affiliations:** 1 Department of Physiology, School of Medicine, and Centro Interdisciplinario de Estudios del Sistema Nervioso (CISNe), Universidad Austral de Chile, Valdivia, Chile; 2 Department of Anatomy, Histology and Pathology, School of Medicine, and Centro Interdisciplinario de Estudios del Sistema Nervioso (CISNe), Universidad Austral de Chile, Valdivia, Chile; University of Nebraska Medical Center, UNITED STATES

## Abstract

Golgi phosphoprotein 3 (GOLPH3) has been implicated in the development of carcinomas in many human tissues, and is currently considered a bona fide oncoprotein. Importantly, several tumor types show overexpression of GOLPH3, which is associated with tumor progress and poor prognosis. However, the underlying molecular mechanisms that connect GOLPH3 function with tumorigenicity are poorly understood. Experimental evidence shows that depletion of GOLPH3 abolishes transformation and proliferation of tumor cells in GOLPH3-overexpressing cell lines. Conversely, GOLPH3 overexpression drives transformation of primary cell lines and enhances mouse xenograft tumor growth *in vivo*. This evidence suggests that overexpression of GOLPH3 could result in distinct features of GOLPH3 in tumor cells compared to that of non-tumorigenic cells. GOLPH3 is a peripheral membrane protein mostly localized at the *trans*-Golgi network, and its association with Golgi membranes depends on binding to phosphatidylinositol-4-phosphate. GOLPH3 is also contained in a large cytosolic pool that rapidly exchanges with Golgi-associated pools. GOLPH3 has also been observed associated with vesicles and tubules arising from the Golgi, as well as other cellular compartments, and hence it has been implicated in several membrane trafficking events. Whether these and other features are typical to all different types of cells is unknown. Moreover, it remains undetermined how GOLPH3 acts as an oncoprotein at the Golgi. Therefore, to better understand the roles of GOLPH3 in cancer cells, we sought to compare some of its biochemical and cellular properties in the human breast cancer cell lines MCF7 and MDA-MB-231 with that of the non-tumorigenic breast human cell line MCF 10A. We found unexpected differences that support the notion that in different cancer cells, overexpression of GOLPH3 functions in diverse fashions, which may influence specific tumorigenic phenotypes.

## Introduction

Compelling experimental evidence indicates that intracellular membrane trafficking factors play important roles in tumorigenesis [1]. For instance, in several human cancers it has been found deregulation of several members of the Rab family of GTP binding proteins and their effectors, which are key regulators in the endocytic and secretory pathways [2]. One such putative membrane trafficking regulator is the protein Golgi phosphoprotein 3 (GOLPH3). In humans, GOLPH3 is encoded in the chromosomal region 5p13, which is a region that is amplified in many solid tumors [3]. In fact, a combination of integrative genomics and clinicopathological and functional analyses have recognized GOLPH3 as an oncoprotein [3]. However, in contrast to other products of oncogenes, GOLPH3 localizes mostly in the Golgi apparatus, and for this reason it has been defined as the first Golgi oncoprotein [3]. Since then, an ever increasing number of reports have been shown that GOLPH3 is overexpressed in several tumor types including bladder cancer, breast cancer, colorectal cancer, esophageal squamous cell carcinoma, gastric cancer, glioma, hepatocellular carcinoma, lung cancer, melanoma, pancreatic ductal adenocarcinoma, prostate cancer, renal cell carcinoma, rhabdomyosarcoma, oral tongue cancer, and epithelial ovarian carcinoma [3–25]. In addition, because in the same tumor types the high expression of GOLPH3 is correlated with poor survival, it has been suggested that the levels of GOLPH3 could be used as biomarker of tumor progression [4–25]. On the other hand, the role of GOLPH3 as an oncoprotein is highlighted by experiments showing that its depletion abolishes transformation and tumor cell proliferation in GOLPH3-overexpressing cell lines, and conversely, that its overexpression drives transformation of primary cell lines and enhances mouse xenograft tumor growth *in vivo* [3]. Interestingly, the oncogenicity of GOLPH3 seems to be mediated by a mechanism that involves enhanced signaling through the mammalian target of rapamycin (mTOR), conferring cancer cells hypersensitivity to rapamycin [3]. To date, however, it remains undetermined how GOLPH3 acts as an oncoprotein at the Golgi [26]. This uncertainty is mainly due to the multiple cellular functions attributed to GOLPH3. In addition to initial studies indicating that GOLPH3 is important for Golgi structure and function [27–30], including sorting of Golgi glycosyltransferases [31–35], later studies suggest functions less conventional for a Golgi protein, such as regulation of cell migration [12,18,25], regulation of cytokinesis [36], regulation of cell survival after DNA damage [37], and even a more *sui generis* function for a Golgi protein, namely the modulation of mitochondrial function [38–40]. As a corollary, GOLPH3 could be mediating several specific functions in different tumor cells, yet little is known about the precise molecular mechanisms and the contribution of these functions to tumorigenesis.

GOLPH3, also referred as GMx33α, GOPP1, GPP34 or MIDAS, or Vps74 in *Saccharomyces cerevisiae*, is a highly conserved phosphoprotein of the Golgi apparatus first found in Golgi proteomic analyses [27,41]. While the genome of invertebrates encode a single GOLPH3 protein, the genome of all vertebrates encodes a second gene corresponding to the paralogue GOLPH3L, also referred as GPP34R or GMx33β [27,41]. In contrast to GOLPH3, GOLPH3L is largely uncharacterized, and although human GOLPH3 and GOLPH3L are 78% similar (65% identical), it seems that GOLPH3L antagonizes the functions of GOLPH3 [42]. GOLPH3 is enriched at the *trans*-Golgi network (TGN) [27], and its association with Golgi membranes, as well as of Vps74 from *Saccharomyces cerevisiae*, depends on its binding to phosphatidylinositol-4-phosphate (PtdIns-4-P) [29,30]. Similar to many membrane trafficking factors, GOLPH3 is a peripheral membrane protein with a large cytosolic pool [27]. It has been shown that GOLPH3 rapidly exchanges between cytosolic and Golgi-associated pools, that it associates with vesicles and tubules arising from the Golgi, and that it can also localize to other cell compartments that include endosomes and the cell surface [28]. This suggests that GOLPH3 could be involved in different membrane trafficking events. Therefore, to understand the multiple roles that the overexpression of GOLPH3 might have in oncogenesis, in this study we aimed to compare GOLPH3 in the human breast cancer cell lines MCF7 and MDA-MB-231, and in the non-tumorigenic, human breast cell line MCF 10A. We found unexpected differences that suggest that the function of GOLPH3 in different tumor cells is more complex than anticipated.

## Materials and Methods

### Cell culture

NRK (normal rat kidney fibroblasts), HeLa (human epithelial), MDA-MB-231 (human breast adenocarcinoma), MCF 10A (human fibrocystic disease), and MCF7 (human breast adenocarcinoma) cells were obtained from the American Type Culture Collection (Manassas, VA). NRK, HeLa and MCF7 cells were maintained in Dulbecco’s Modified Eagle’s Medium (DMEM; Life Technologies). MCF 10A and MDA-MB-231 cells were maintained in DMEM-F12 (Life Technologies). For all cell lines the media were supplemented with 10% heat-inactivated fetal bovine serum, 100 U/ml penicillin, 100 μg/ml streptomycin (Life Technologies), and 5 μg/ml plasmocin (InvivoGen, San Diego, CA). MCF 10A and MCF7 media were further supplemented with 10 μg/ml insulin (Sigma-Aldrich). MCF 10A medium was also supplemented with 100 ng/ml cholera toxin, 20 ng/ml EGF, and 0.5 μg/ml hydrocortisone (Sigma-Aldrich).

### Antibodies

We used the following mouse monoclonal antibodies: clone AC-74 to β-actin (Sigma-Aldrich), clone 35/GM130 to GM130 (BD Biosciences), clone 464.6 to Sodium Potassium-ATPase α 1 subunit (Abcam), and Horseradish Peroxidase-conjugated IgG to green fluorescent protein (Miltenyi Biotec; cat # 130-091-833). We used polyclonal antibodies to the following proteins: β-COP (Affinity Bioreagents; cat # PA1-061), GOLPH3 (Abcam; cat # ab98023), Syntaxin 16 (Synaptic Systems; cat # 110162), TGN38 (AbD Serotec; cat # AHP499G) and TGN46 (AbD Serotec; cat # AHP500G). The following fluorochrome-conjugated antibodies were from Life Technologies: Alexa Fluor-488–conjugated donkey anti mouse IgG, Alexa Fluor-594–conjugated donkey anti rabbit IgG, Alexa Fluor-647-conjugated donkey anti sheep IgG, and Alexa Fluor-647-conjugated donkey anti mouse IgG. HRP-conjugated secondary antibodies were from Jackson ImmunoResearch. Depending on their reactivity, primary antibodies were used at a dilution 1/200 to 1/2000. HRP-conjugated secondary antibodies were used at dilutions 1/1000 to 1/20000, depending on their reactivity. All Alexa Fluor-conjugated secondary antibodies were used at a dilution 1/1000.

### Subcellular Fractionation, Membrane Fractions Stripping and Size Exclusion Chromatography

Golgi and cytosolic fractions from rat liver were prepared as described [28,43]. For isolation of membranes and cytosol from cultured cells we used a similar method described previously [28]. All the procedures were performed at 4°C. Briefly, untreated or treated cells grown in 100-mm diameter culture dishes were scraped with a rubber policeman in 1 ml of homogenization buffer (0.1 M KH_2_PO_4_, 0.1 M K_2_HPO_4_, 5 mM MgCl_2_, 0.25 M sucrose, pH 7.4) supplemented with a cocktail of protease inhibitors (416 μM 4-(2-aminoethyl) benzenesulfonyl fluoride hydrochloride, 0.32 μM aprotinin, 16 μM bestatin, 5.6 μM E-64, 8 μM leupeptin, 6 μM pepstatin A; Sigma-Aldrich), and a cocktail of phosphatase inhibitors (1 mM NaF, 0.3 mM Na_2_P_2_O_7_, 1 mM Na_3_VO_4_; Sigma-Aldrich). Homogenization was done by passing the suspension of scraped cells 30 times through a ball-bearing cell-cracker (EMBL, Heidelberg), and the homogenate was spun at 1,000 x g for 10 min. The post-nuclear supernatant (700 μl) was centrifuged at 100,000 x g for 15 min. The supernatant, designated as cytosol, was collected and analyzed immediately. The pellet was washed once with homogenization buffer and centrifuged as before. The second supernatant was discarded and the pellet was resuspended in 300 μl of homogenization buffer, and analyzed immediately. After protein concentration estimation, aliquots of both cytosol and membranes were stored at -80°C for further analysis. For stripping of peripheral proteins, membrane fractions were prepared as described above, resuspended in 10 mM Tris HCl pH 7.4, and divided into equal parts of 35 μl that were incubated on ice for 1 hour with 700 μl of either 10 mM Tris HCl pH 7.4 (control stripping), 1 M KCl in 10 mM Tris HCl pH 7.4 (salt stripping) or 0.2 M Na_2_CO_3_ pH 11.3 (high pH stripping). Stripped membrane fractions were pelleted, and extracted proteins in the supernatants were precipitated with 10% trifluoroacetic acid at 4°C and collected by centrifugation. Pelleted membranes were washed once with 700 μl of the corresponding stripping solution followed by centrifugation. Pellets containing proteins were processed with Laemmli sample buffer [44] for further SDS-PAGE analysis. Size exclusion chromatography of cytosolic samples was performed on a Superose 6 10/300 GL column (GE Healthcare), equilibrated in 50 mM Tris HCl, and 0.5 M NaCl, pH 8.0. Stokes radii (in Å) were estimated using the following proteins: bovine thyroid thyroglobulin (85.0 Å), horse spleen ferritin (61.0 Å), bovine liver catalase (52.2 Å), bovine serum albumin (35.5 Å), and bovine pancreas ribonuclease A (16.4 Å) (GE Healthcare).

### Preparation of Protein Extracts, Protein Electrophoresis and Immunoblotting

Protein extract preparation, SDS-PAGE analysis and immunoblotting were performed as described [28,45,46].

### Immunofluorescence Microscopy and Quantitative Analysis

Cells left untreated or treated with 5 μg/ml Brefeldin A (BFA) for 60 min at 37°C were analyzed by immunofluorescence microscopy as described previously [47]. Fixation of cells with methanol or 4% paraformaldehyde was performed when primary antibody reactivity demanded. Fluorescence microscopy images were acquired with an AxioObserver.D1 microscope equipped with a PlanApo 63x oil immersion objective (NA 1.4), and an AxioCam MRm digital camera (Carl Zeiss), using similar settings as described previously [46]. To prepare figures, images were processed with Image J software (version 1.44o; Wayne Rasband, NIH, http://imagej.nih.gov) and Adobe Photoshop CS3 software (Adobe Systems, Mountain View, CA). Analysis and quantification of fluorescent signal, and estimation of the Pearson's correlation coefficient, *r* [48], were performed as described [49].

### Recombinant cDNA Constructs and Transfection

For the generation of GOLPH3 constructs, a cDNA encoding full-length human GOLPH3 (GenBank/EMBL/DDBJ accession number NM_022130) was acquired from OriGene Technologies (Rockville, MD), and used as a template. Full-length GOLPH3 was obtained by PCR amplification and cloned in-frame into the *Eco*RI and *Sal*I sites of the pEGFP-C2 vector (Takara Clontech), or into the *Eco*RI and *Sal*I sites of the pGST-Parallel-1 vector [50]. We also used plasmids encoding the following proteins tagged with the green fluorescent protein (GFP): ε-COP-GFP [51], GFP-Rab6A [52], GFP-GGA1 [53], GFP-golgin-84 [54], and GFP-GRASP55 [55]. The nucleotide sequence of all recombinant constructs was confirmed by dideoxy sequencing. Transient transfections were carried out using the Lipofectamin 2000 reagent (Life Technologies) according to the manufacturer's instructions, and the cells were analyzed 14–24 h after transfection.

### Time-Lapse Microscopy and Image Analysis

Time-lapse fluorescence imaging was as described [47]. Briefly, live cells grown on 35-mm glass-bottom culture dishes (MatTek) were transfected to express GFP-tagged proteins (GFP-GOLPH3, ε-COP-GFP, GFP-Rab6A, or GFP-GGA1). After 14–18 hours, the normal culture medium was replaced by phenol red-free buffered medium, and culture dishes with cells were held at 37°C on a LU-CB1 Leiden Micro-Incubator (Harvard Apparatus, Holliston, MA) attached to a temperature controller (Medical Systems Corp., Greenvale, NY). Images were acquired with an AxioObserver.D1 microscope equipped with a PlanApo 63x oil immersion objective (NA 1.4) and an AxioCam MRm digital camera using AxioVision software (Carl Zeiss). Images were processed and converted to Quicktime movies with Image J software (version 1.44o). Depending on the GFP-tagged protein, quantification of tubule-vesicular profiles was performed by visual inspection of movies prepared with images acquired during 20–180 seconds. To prepare figures, single frames were processed with Adobe Photoshop CS3 software. For quantitative photobleaching, laser scanning confocal microscopy images were acquired with an Olympus FluoView FV1000 scanning unit fitted on an inverted Olympus IX81 microscope equipped with a PlanApo 60x oil immersion objective (NA 1.4; Olympus), using similar settings as described previously [28]. Quantitative analysis of images was performed with Image J software (version 1.44o), as described [45]. For quantitative analysis of fluorescence recovery after photobleaching (FRAP), the fitting of the data was performed with SigmaPlot software (version 12.5; Systat Software Inc.), and was used to obtain the percentage of maximal fluorescence recovery and the halftime of maximal fluorescence recovery (*t*_1/2_) of at least ten FRAP experiments on each cell line. To prepare figures, single frames were also processed with the software Adobe Photoshop CS3.

### Two-dimensional Gel Electrophoresis and Phosphatase Assay

For two-dimensional gel electrophoresis (2-D GE), protein samples (30–100 μg) were precipitated using 2-D Clean-Up Kit (GE Healthcare) according to the manufacturer's instructions. Precipitated proteins were solubilized in isoelectric focusing (IEF) solution (7 M urea, 2 M thiourea, 2% CHAPS, 0.002% bromophenol blue, 0.5% IPG buffer pH 3–10 NL; GE Healthcare). The first dimension consisted of IEF performed using 7-cm dry strip gels (Immobiline DryStrip gels, nonlinear pH range 3–10; GE Healthcare). The dry strip gels were rehydrated with the solubilized protein sample (130 μl) by in-gel reswelling on an Immobiline DryStrip IPGbox (GE Healthcare) at 20°C for 16 hours. Rehydrated strip gels were transferred to an Ettan IPGphor 3 Manifold (GE Healthcare), and IEF was allowed to proceed for a maximum of 7000 V and 50 μA per strip, at 20°C for 12 hours (16 kVh). In parallel, we processed proteins used as IEF markers (isoelectric points, pI, in parenthesis): *Aspergillus niger* amyloglucosidase (pI = 3.6), bovine β-lactoglobulin A (pI = 5.1), bovine carbonic anhydrase II (pI = 5.9), and horse heart myoglobin (6.8, 7.2) (Sigma-Aldrich). Immobiline strip gels were incubated in SDS equilibration buffer solution (6 M urea, 75 mM Tris HCl, 30% glycerol, 2% SDS, 0.002% bromophenol blue, pH 8.8) supplemented with 10 mg/ml DTT, at 20°C for 10 min with constant agitation, followed by a similar incubation, but with SDS equilibration buffer solution supplemented with 25 mg/ml iodoacetamide. The second dimension consisted of SDS-PAGE, followed by immunoblot with antibody to GOLPH3. For dephosphorylation prior to 2-D GE, a sample of rat liver Golgi membranes, and of cytosolic and membrane fractions of each cell line (100 μg of proteins), was incubated with calf intestinal alkaline phosphatase (New England BioLabs) according to the manufacturer's instructions. Proteins were precipitated and processed for 2-D GE as described above.

### Expression and Purification of Recombinant GOLPH3, and Lipid-binding Assay

Recombinant GOLPH3 tagged with an N-terminal glutathione S-transferase (GST) followed by a tobacco etch virus (TEV) protease cleavage site was expressed and purified using a similar method described previously [46], with minor modifications. Briefly, expression in *E*. *coli* B834(DE3) (Novagen, Madison, WI) was induced with 0.5 mM IPTG at 25°C for 16 hours. Pellets of bacteria were resuspended in homogenization buffer (50 mM Tris HCl, 0.5 M NaCl, 10% glycerol, 5 mM β-mercaptoethanol, and 2 mM phenylmethylsulfonyl fluoride, pH 8.0), and lysed by sonication. The clarified supernatant was purified on glutathione-Sepharose 4B (GE Healthcare). After removal of the GST moiety by TEV cleavage, and sequential passage through glutathione-Sepharose 4B and Ni-NTA (QIAGEN) resins, GOLPH3 was further purified on a Superdex 200 column (GE Healthcare). For lipid binding, membranes with spotted phospholipids (Echelon Biosciences) were blocked in 0.2% fatty acid-free BSA in blocking buffer (25 mM Tris HCl, 150 mM NaCl, 1 mM DTT, pH 7.4) at 20°C for 2 hours with constant agitation. Recombinant GOLPH3 (300 μg) was either left untreated or mixed with cytosolic proteins from cultured cells (1 mg), followed by incubation in 3 ml of binding buffer (25 mM Tris HCl, 150 mM NaCl, 0.2% fatty acid-free BSA, 1 mM DTT, 0.01% Tween 20, pH 7.4; supplemented with a cocktail of protease inhibitors and a cocktail of phosphatase inhibitors described above) at 20°C for 15 min. Membranes with spotted phospholipids were blotted with untreated or cytosol-incubated GOLPH3 in binding buffer at 4°C for 16 hours with constant agitation. The membranes were washed 3 times in 10 ml of washing buffer (25 mM Tris HCl, 150 mM NaCl, 1 mM DTT, 0.01% Tween 20, pH 7.4) at 20°C for 15 min, followed by immunoblot with antibody to GOLPH3. As a control, membranes with spotted lipids were incubated as described above, but with only the cytosolic proteins from cultured cells (1 mg), followed by immunoblot with antibody to GOLPH3.

### Densitometric Quantification and Statistical Analysis

The amount of immunoblot signal from images with unsaturated pixels was estimated using Image J software (version 1.44o). For each condition, protein bands were quantified from at least three independent experiments. Statistical analysis was performed using Microsoft Excel for Mac 2011 (Microsoft Corporation). When appropriate, results are represented in graphs depicting the mean ± standard deviation. Statistical significance was determined by two-tailed, paired *t*-test. *P*-values > 0.05 or *≤* 0.05 were regarded as not statistically significant or statistically significant, respectively. In the figures, *P*-values between 0.01 and 0.05 are indicated with one asterisk, *P*-values between 0.001 and 0.01 are indicated with two asterisks, and *P*-values less than 0.001 are indicated with three asterisks.

## Results and Discussion

### GOLPH3 distributes differently in cytosolic and membrane-bound pools in different human breast cell lines

**Fig 1:**
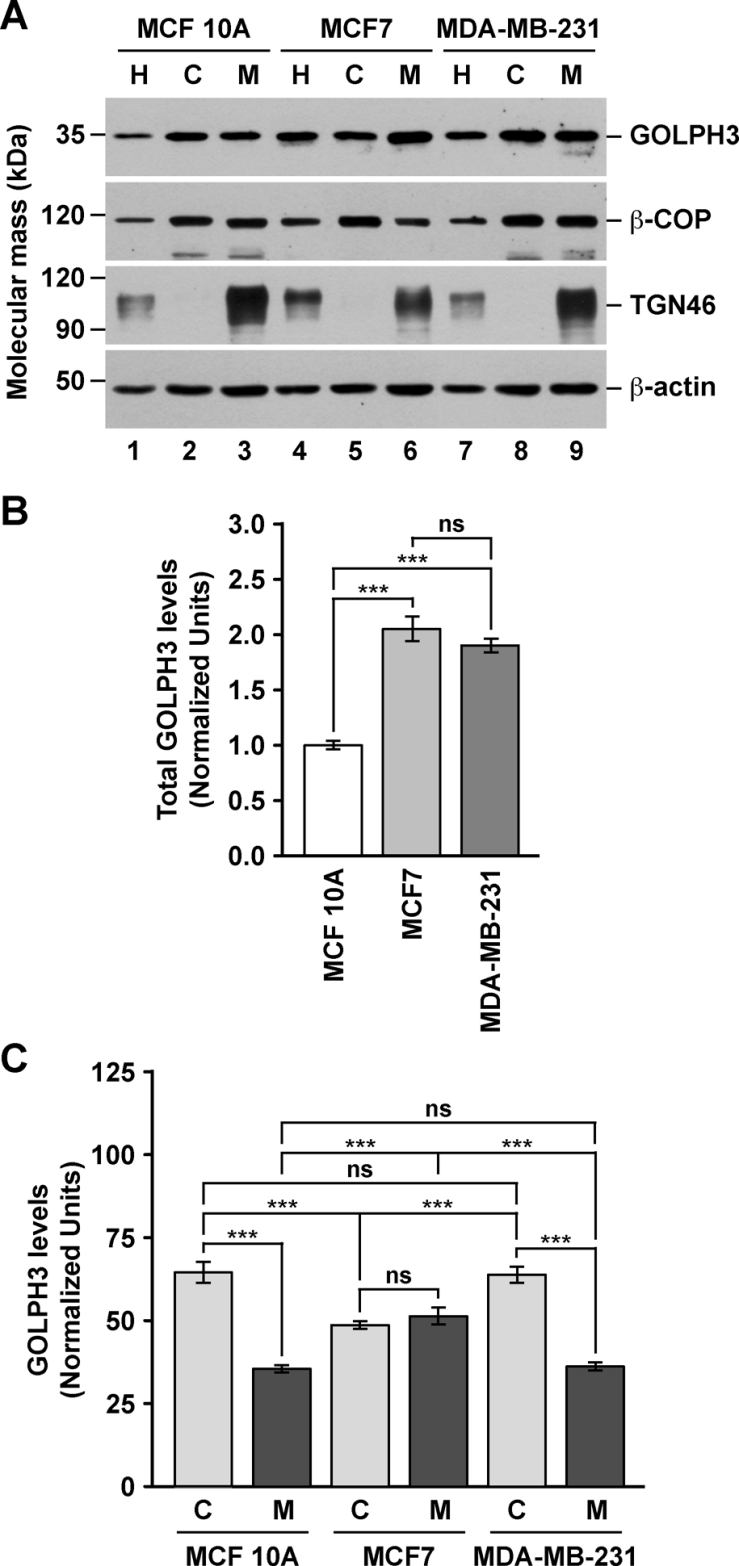
The levels and subcellular distribution of GOLPH3 are different in different human breast cell lines. (A) Cell homogenates (*H*) from the indicated cell lines were used to prepare cytosolic (*C*) and membrane (*M*) fractions. Equivalent amounts of each fraction (10 μg of proteins) were subjected to SDS-PAGE and immunoblotting using antibodies to the proteins indicated on the right. The position of molecular mass markers is indicated on the left. (B) Densitometric quantification of the immunoblot signal of the levels of GOLPH3 in the cell homogenates as shown in (A). (C) Densitometric quantification of the immunoblot signal of the levels of GOLPH3 in cytosolic (*C*) and membrane (*M*) fractions as shown in (A). Bar represents the mean ± standard deviation of the amount of immunoblot signal normalized with the signal for β-actin, and also for the total amount of protein in each fraction (for more details see S1 Table). *** *P* < 0.001; *ns*, not statistically significant.

**Fig 2:**
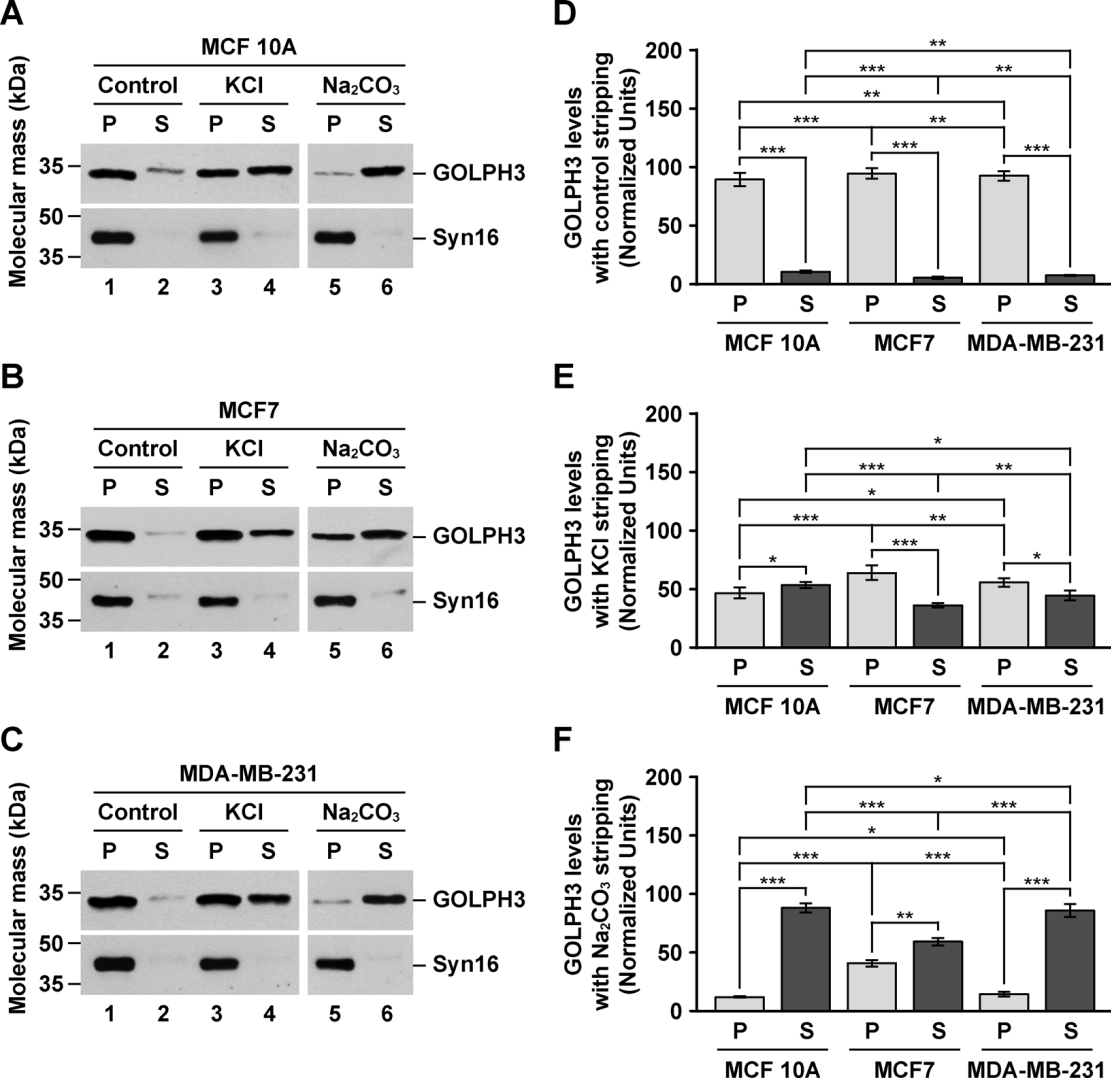
GOLPH3 from MCF7 cells is more tightly bound to membranes than in MCF 10A or MDA-MB-231 cells. (A-C) Samples of a membrane fraction (70 μg of proteins) from MCF 10A (A), MCF7 (B), and MDA-MB-231 (C) cells were incubated on ice for 1 hour with either 10 mM Tris HCl pH 7.4 (*Control*), 1 M KCl in 10 mM Tris HCl pH 7.4 (*KCl*) or 0.2 M Na_2_CO_3_ pH 11.3 (*Na*_*2*_*CO*_*3*_). After centrifugation, pelleted membranes (*P*) and extracted proteins in the supernatant (*S*) were processed by SDS-PAGE and immunoblotting using antibodies to the proteins indicated on the right. *Syn16*, Syntaxin 16. The position of molecular mass markers is indicated on the left. (D-F) Densitometric quantification of the immunoblot signal of the levels of GOLPH3 in pellets (*P*) and supernatants (*S*) as shown in A-C of membranes incubated in control conditions (D), in 1 M KCl (E), or in 0.2 M Na_2_CO_3_ (F). Bar represents the mean ± standard deviation of the amount of immunoblot signal. * *P* < 0.05; ** *P* < 0.01; *** *P* < 0.001.

**Fig 3:**
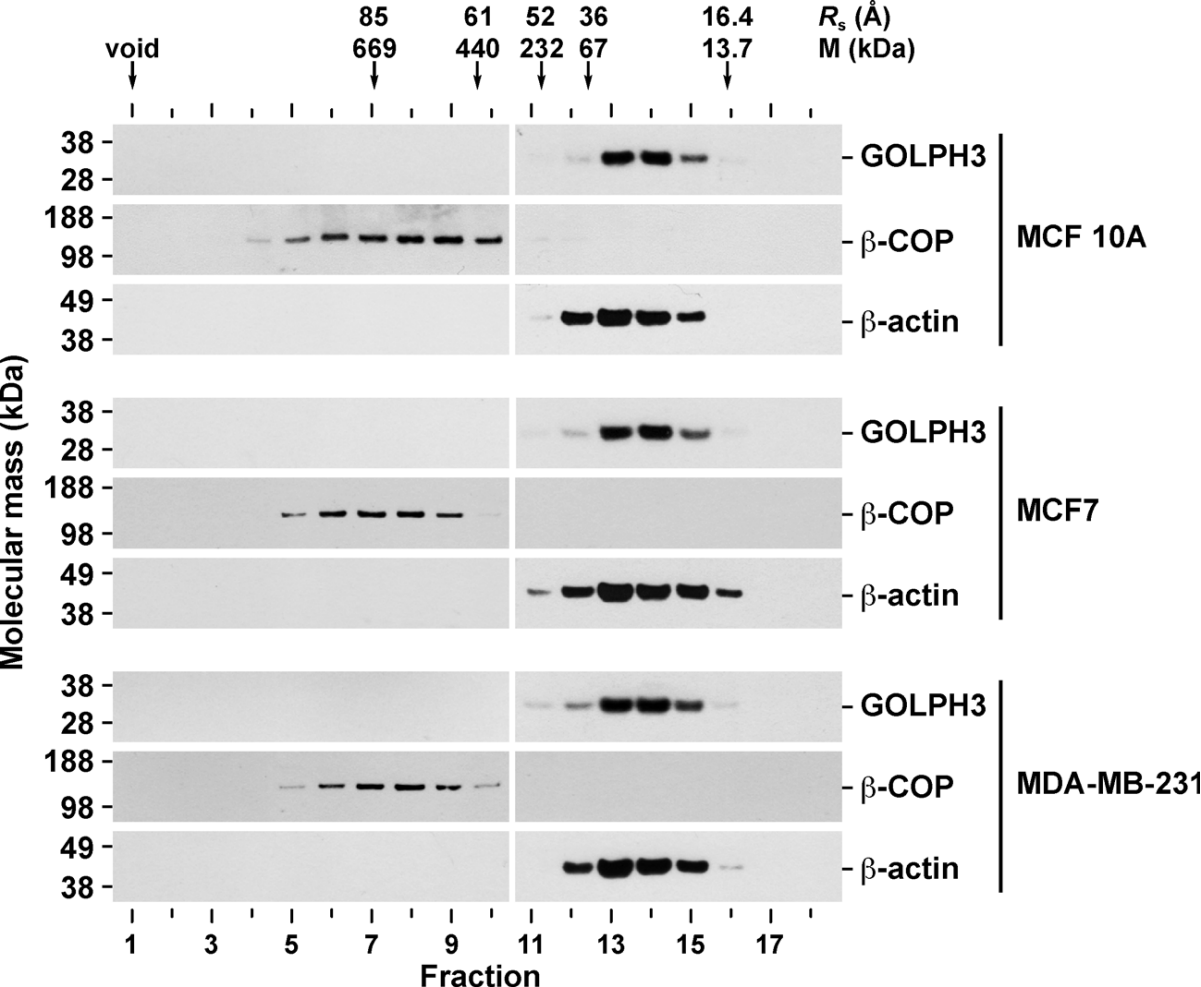
Cytosolic GOLPH3 behaves as a monomer in MCF 10A, MCF7, and MDA-MB-231 cells. Samples of a cytosolic fraction from the indicated cell lines were analyzed by gel filtration on a Superose 6 column. Arrows indicate the elution position of molecular size markers. Stokes radii (*R*_s_) are indicated in Angstroms (*Å*). As a reference, the molecular mass (*M*) of molecular size markers is also indicated. Fractions were subjected to SDS-PAGE and immunobloting with antibodies to the proteins indicated on the right. The position of molecular mass markers is indicated on the left.

Numerous reports have shown that GOLPH3 is overexpressed in a variety of human tumor tissues, including breast cancer [3–25]. Likewise, it has been shown that the levels of GOLPH3 are higher in breast cancer cell lines compared to that of normal breast cells [9]. We hypothesized that in cancer cells, increased levels of GOLPH3 results in altered cellular functions as a consequence of biochemically distinct pools of GOLPH3 that otherwise are absent or in low concentration in normal cells. To analyze this possibility, we studied GOLPH3 in the estrogen receptor positive (ER+) MCF7 cells, the estrogen receptor negative (ER-) MDA-MB-231 cells, and the non-tumorigenic MCF 10A cells. It has been shown that in both rat liver tissue and normal rat kidney (NRK) cells, GOLPH3 is found bound to membranes, but also contained in a cytosolic pool [27,28]. Thus, we first evaluated the subcellular distribution of GOLPH3 by immunoblot analysis of cytosolic and membrane fractions obtained by differential centrifugation. Unexpectedly, we found that the total levels of GOLPH3 in MCF7 and MDA-MB-231 cells are around twice as high as they are in MCF 10A cells (Fig 1A, lanes 1, 4 and 7, Fig 1B and S1 Table). When we analyzed the same amount of protein from each fraction, we found that, in contrast to the Golgi transmembrane protein TGN46, GOLPH3 distributed also in the cytosol of each cell line, similar to the Golgi peripheral membrane protein β-COP (Fig 1A). To have a better quantitative assessment of GOLPH3 partitioning, we estimated the amount of GOLPH3 relative to the total protein content obtained in each fraction. We found that in the three cell lines the relative amount of cytosolic and membrane proteins were similar, with 59.7 ± 0.2%, 61.4 ± 0.6% and 61.7 ± 1.0% of proteins partitioned in the cytosolic fraction of MCF7, MDA-MB-231, and MCF 10A cells, respectively (S1 Table). However, the proportion of GOLPH3 between the cytosolic and membrane fractions was not the same among cell lines. While the cytosol to membrane ratio of GOLPH3 was ∼1.8 in both MCF 10A (64.5 ± 3.2% to 35.5 ± 1.1%) and MDA-MB-231 (63.8 ± 2.5% to 36.2 ± 1.2%) cells (S1 Table and Fig 1A, lanes 2, 3, 8 and 9, and Fig 1C), in MCF7 cells the ratio was ∼0.9 (48.7 ± 1.2% to 51.3 ± 2.5%; S1 Table and Fig 1A, lanes 5 and 6, and Fig 1C). This significant difference suggests that GOLPH3 in MCF7 cells is contained in distinct membrane pools. Alternatively, GOLPH3 could be more tightly bound to membranes in MCF7 cells. To distinguish between these two possibilities, we performed membrane stripping with 1 M KCl (salt stripping) or 0.2 M Na_2_CO_3_ pH 11.3 (high pH stripping), treatments that result in extraction of GOLPH3 from both rat liver Golgi membranes and a membrane fraction of NRK cells [28]. Immunoblot analysis with antibody to the Golgi transmembrane protein syntaxin 16 showed that the integrity of membranes seems well preserved with either of the stripping procedures (Fig 2A–2C). Interestingly, control stripping resulted in extraction of GOLPH3 from membranes, although to a low degree (Fig 2A–2C, lanes 1 and 2). Nevertheless, the amount of GOLPH3 extracted in control conditions showed significant differences between the three cell lines (Fig 2D), being slightly less sensitive to extraction in MCF7 cells (5.4 ± 1.1%) as compared to MDA-MB-231 cells (7.4 ± 0.4%) or MCF 10A cells (10.6 ± 1.0%). This assumption was corroborated with salt stripping, showing that only 36.1 ± 2.0% of GOLPH3 was extracted from membranes of MCF7 cells compared to 44.5 ± 4.4% from membranes of MDA-MB-231 cells and 53.3 ± 2.6% from that of MCF 10A cells (Fig 2A–2C, lanes 3 and 4, and Fig 2E). High pH stripping provided additional evidence that GOLPH3 is differentially bound to membranes in these cell lines. As expected for a peripheral membrane protein [56], in this condition the majority of GOLPH3 was stripped from membranes of MCF 10A cells (88.0 ± 3.9%; Fig 2A, lanes 5 and 6, and Fig 2F), and of MDA-MB-231 cells (85.7 ± 5.6%; Fig 2C, lanes 5 and 6, and Fig 2F). In contrast, significantly less GOLPH3 (59.2 ± 3.3%) was extracted from membranes of MCF7 cells (Fig 2B, lanes 5 and 6, and Fig 2F). Further analysis showed that, in fact, the differences among the three cell lines in sensitivity to both salt and high pH stripping were significant (Fig 2E and 2F). These data suggest that the association of GOLPH3 to membranes could be differentially regulated, not only between non-tumorigenic cells and cancer cells, but also between different types of cancer cells. One possible explanation of these differences could be the oligomeric state of cytosolic GOLPH3. Crystallographic data shows that GOLPH3 forms a crystallographic dimer [30], and that its yeast orthologue Vps74 forms a crystallographic tetramer [31]. Thus, it seems plausible that the high levels of GOLPH3 in cancer cells could result in stabilization of a cytosolic oligomeric form. To test this possibility, we analyzed the cytosolic fraction of the three cell lines by size exclusion chromatography followed by immunoblotting. As controls, we performed immunoblotting to monomeric actin, as well as to the heptameric, Golgi coat protein complex I (COPI) detected with antibody to the β-COP subunit [57]. Based on the hydrodynamic properties of the oligomeric (and monomeric) proteins used to calibrate the chromatography column, we found that, in contrast to our hypothesis, cytosolic GOLPH3 from each cell line behaved as a fairly globular, monomeric protein, in clear contrast to β-COP (Fig 3). Moreover, the peak of diffusion of GOLPH3 (M = 34 kDa; fraction 14) is unambiguously distinguishable from that of actin (M = 42 kDa; fraction 13) (Fig 3), indicating that in these cell lines cytosolic GOLPH3 exists largely as a monomer. This result suggests that (a) different feature(s) of cytosolic GOLPH3 should account for the differences that we found in membrane association.

### GOLPH3 from MCF7 cells has a distinct sensitivity to Brefeldin A

**Fig 4:**
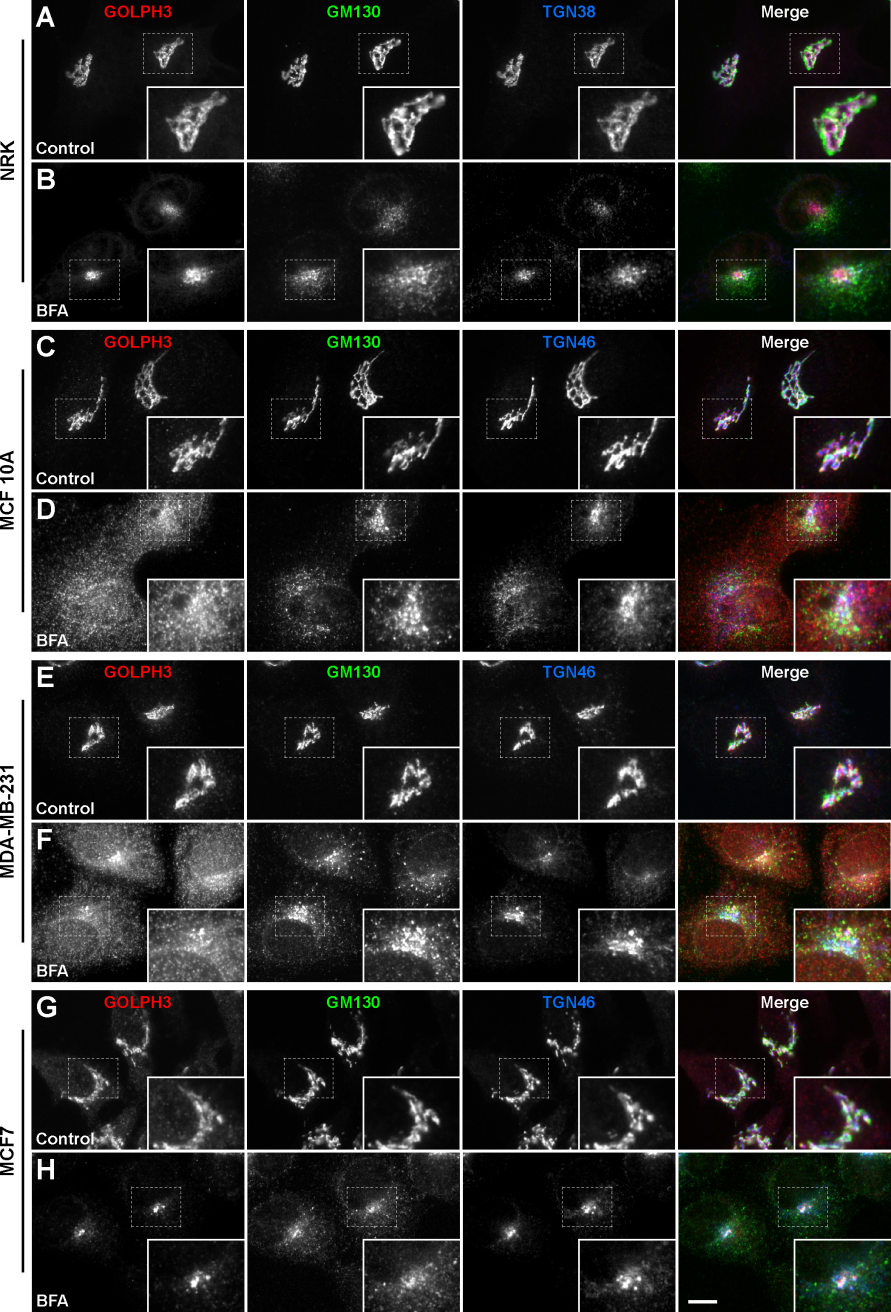
The sensitivity of GOLPH3 to BFA is different in different human breast cell lines. NRK (A and B), MCF 10A (C and D), MDA-MB-231 (E and F), and MCF7 (G and H) cells were left untreated (*Control*) or treated with 5 μg/ml BFA for 60 min (*BFA*). Cells were fixed, permeabilized, and immunolabeled with rabbit polyclonal antibody to GOLPH3, mouse monoclonal antibody to GM130, and either sheep antibody to TGN38 (A and B) or sheep antibody to TGN46 (C to H). Secondary antibodies were Alexa-594-conjugated donkey anti-rabbit IgG (red channel), Alexa-488-conjugated donkey anti-mouse IgG (green channel), and Alexa-647-conjugated donkey anti-sheep IgG (blue channel). Stained cells were examined by fluorescence microscopy. Merging red, green, and blue channels generated the fourth image on each row; yellow indicates overlapping localization of the red and green channels, cyan indicates overlapping localization of the green and blue channels, magenta indicates overlapping localization of the red and blue channels, and white indicates overlapping localization of all three channels. Insets show 1.7x magnifications. Bar, 10 μm.

Electron and fluorescence microscopy analyses show that GOLPH3 in NRK cells is localized mostly at the TGN where it co-localizes with the TGN marker TGN38 [27]. Thus, we next compared the subcellular distribution of GOLPH3 by fluorescence microscopy. The overall appearance of the immunofluorescence signal indicated that, as in NRK cells (Fig 4A), the localization of GOLPH3 in the three human breast cell lines is also restricted to the Golgi apparatus, as assessed by co-labeling with antibodies either to the *cis*-Golgi marker GM130 or to the TGN marker TGN46 (Fig 4C, 4E and 4G). Intriguingly, we observed that, in contrast to NRK cells, the degree of overlap between the signal of GOLPH3 and that of the TGN marker seemed less prominent in either of the three breast cell lines. We confirmed this assumption by colocalization analysis. We found that while in NRK cells 97.9 ± 0.3% of GOLPH3 overlapped with TGN38 (*r* = 0.983 ± 0.006; Fig 4A), the overlap of GOLPH3 with TGN46 was reduced and varied among the three breast cell lines, being 84.6 ± 2.7% in MCF 10A cells (*r* = 0.882 ± 0.022; Fig 4C), 80.7 ± 0.8% in MDA-MB-231 cells (*r* = 0.892 ± 0.034; Fig 4E), and 93.3 ± 1.1% in MCF7 cells (*r* = 0.920 ± 0.015; Fig 4G). On the other hand, the overlap of GOLPH3 with GM130 was 81.1 ± 1.6% in NRK cells (*r* = 0.787 ± 0.010; Fig 4A), 87.3 ± 1.2% in MCF 10A cells (*r* = 0.852 ± 0.020; Fig 4C), 87.5 ± 1.3% in MDA-MB-231 cells (*r* = 0.838 ± 0.024; Fig 4E), and 82.0 ± 0.7% in MCF7 cells (*r* = 0.874 ± 0.009; Fig 4G). Although a more conclusive analysis of the localization of GOLPH3 would require further characterization by electron microscopy, our colocalization results provide additional support to the notion that the association of GOLPH3 with (Golgi) membranes is distinct among different cell lines, and suggests that the function of GOLPH3 is not restricted to the TGN. In agreement with this possibility, several reports indicate that GOLPH3 plays functional roles in other regions of the Golgi apparatus, such as in *cis*-more cisternae. For instance, several lines of evidence suggest that the mechanism by which GOLPH3 promotes retention of glycosyltransferases at the Golgi is through its interaction with COPI [32,58,59], which mediates vesicular transport within the Golgi cisternae and from the *cis*-Golgi to the endoplasmic reticulum (ER) [60]. This differential distribution of proteins within the Golgi, i.e., *cis* and medial cisternae versus the TGN, is often investigated by evaluating sensitivity to Brefeldin A (BFA). BFA is a fungal metabolite that negatively affects the formation of coated vesicles, such as of COPI-coated vesicles at the Golgi, by means of inhibiting the GTP exchange reactions by a subclass of GTP binding proteins regulatory elements [61]. In fact, an early event in BFA-treated cells is the rapid redistribution into the cytosol of peripherally, membrane-bound β-COP [62]. This and other events eventually have a profound effect on the structure of the Golgi apparatus. During BFA treatment *cis-* and medial-Golgi resident transmembrane proteins [63], as well as Golgi matrix proteins [64], are redistributed to the ER. In contrast, TGN proteins are redistributed toward the microtubule organizing center (MTOC; [65]). Accordingly, BFA treatment in NRK cells results in MTOC redistribution of GOLPH3 together with TGN38 ([27]; Fig 4B). We found that similar BFA treatment resulted in the expected MTOC redistribution of TGN46 in the three breast cell lines (Fig 4D, 4F and 4H). However, we observed that GOLPH3 redistributed mainly to the MTOC only in MCF7 cells (Fig 4H). In MCF 10A and MDA-MB-231 cells, GOLPH3 had also a more diffuse distribution (Fig 4D and 4F), suggesting localization at the ER and/or the cytosol. Immunoblot analysis of membrane and cytosolic fractions of cells treated with BFA confirmed that a fraction of GOLPH3 redistributed to the cytosol, although to a lesser extent in MCF7 cells (S1 Fig). Whether the behavior of GOLPH3 was a consequence of different functional properties of the Golgi apparatus or of different properties of BFA-sensitive regulators, or both, is currently unknown. However, one possible interpretation of these results is that GOLPH3 in MCF7 cells is more tightly bound to Golgi membranes, compared to that in MCF 10A and MDA-MB-231 cells. We hypothesize that a differential regulation of GOLPH3 in these cell lines could also result in distinct effects on the membrane trafficking events in which it participates.

### GOLPH3 has different dynamic behavior in different human breast cell lines

**Fig 5:**
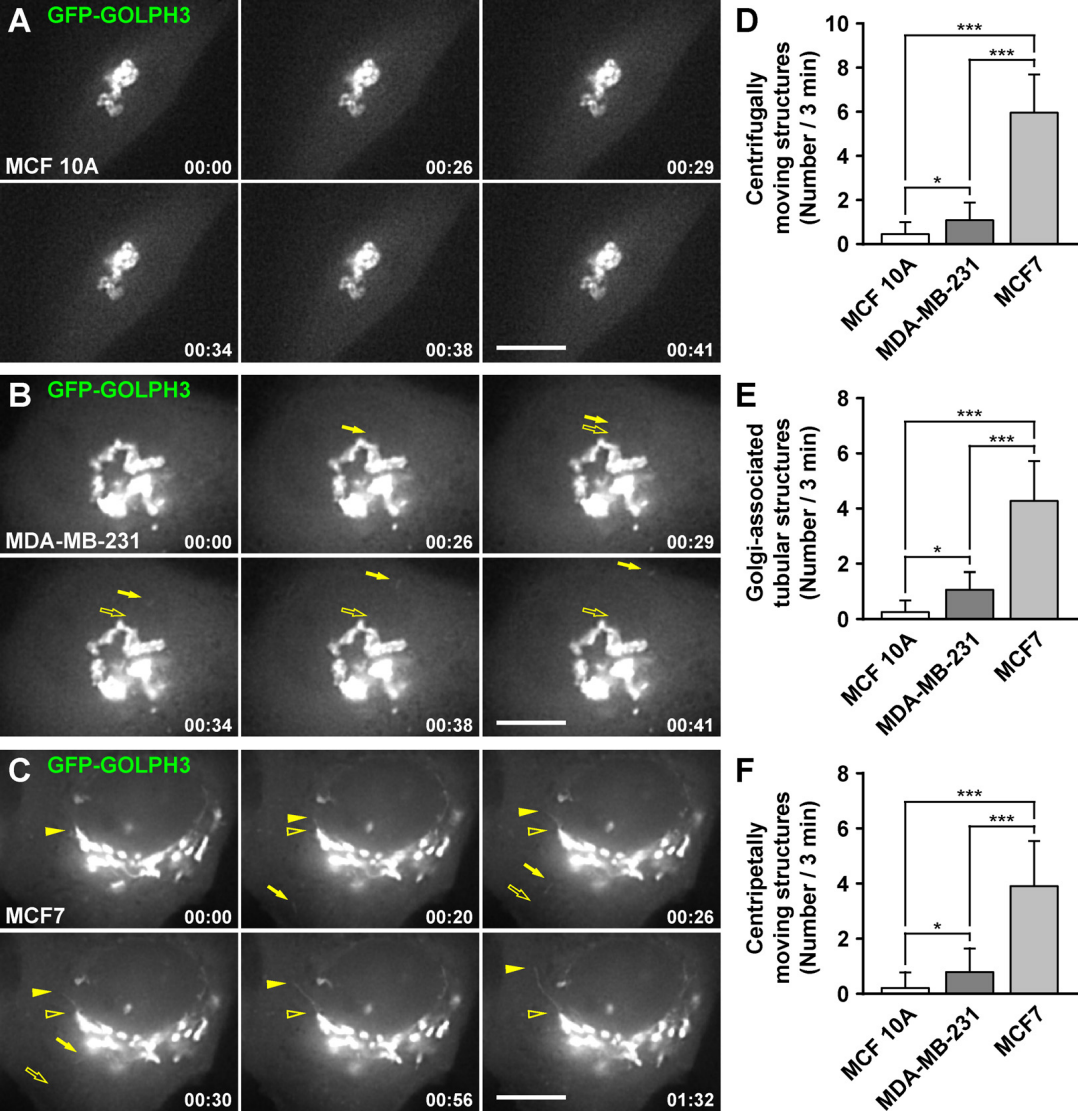
The dynamic behavior of GFP-GOLPH3 is different in different human breast cell lines. (A-C) MCF 10A (A), MDA-MB-231 (B), and MCF7 (C) cells transiently expressing GFP-GOLPH3 were held in a microscope stage at 37°C and examined by fluorescence microscopy. The time after initiation of imaging is shown in the bottom right corner of each panel in minutes:seconds. Images are representative of 15–20 videos of up to 200 seconds of recording. In B, filled arrows indicate a vesicular structure moving from the Golgi to the periphery of the cell. In C, filled arrows indicate a vesicular structure moving from the periphery of the cell to the Golgi area, and filled arrowheads indicate a tubular structure elongating from the Golgi. Empty arrows and empty arrowheads indicate the initial position of mobile structures. Bars, 5 μm. (D-F) The number of tubule-vesicular structures moving centrifugally (D), the number of tubular structures elongating from the Golgi (E), or the number of tubule-vesicular structures moving centripetally (F), were quantified from videos corresponding to 180 seconds of imaging. Bar represents the mean + standard deviation of the observed profiles (n = 15). * *P* < 0.05; *** *P* < 0.001.

To evaluate whether GOLPH3 participates in distinct membrane trafficking events in different cell lines, we transiently expressed GOLPH3 tagged with the green fluorescent protein (GFP-GOLPH3) for analysis in live cells by fluorescence microscopy. It has been shown that in NRK cells, GFP-GOLPH3 is phenotypically indistinguishable from endogenous GOLPH3 [28]. Similarly, we found that GFP-GOLPH3 localized well at the Golgi apparatus in each of the three breast cell lines (S2 Fig), and distributed in both cytosolic and membrane fractions in a manner indistinguishable from that of endogenous GOLPH3 (S3 Fig). Time-lapse imaging in NRK cells has shown that GFP-GOLPH3 emerges from the Golgi in vesicular and tubular structures that detach and move throughout the cell [28]. Strikingly, we observed a very dissimilar behavior of GFP-GOLPH3 among breast cell lines. While in both MCF 10A and MDA-MB-231 cells, few structures containing GFP-GOLPH3 emerged from the Golgi (Fig 5A, 5B, 5D and 5E and S1 and S2 Videos), we frequently observed in MCF7 cells both vesicular and tubular structures moving to the periphery of the cells (Fig 5C, 5D and 5E and S3 Video). Unexpectedly, we also observed vesicular and tubular structures moving from the periphery of the cell to the Golgi region, although significantly more frequently in MCF7 cells (Fig 5C and 5F). This observation, however, is consistent with reports indicating that GOLPH3 plays a role in retrograde trafficking cooperating with the retromer complex [3,66]. To date, it is not clear what the precise role of GOLPH3 is in membrane trafficking events. Although compelling evidence suggests that GOLPH3 and its orthologues collaborate in the sorting of some Golgi glycosyltransferases [31–35], several reports have shown that they also have roles in the trafficking of cargoes out of the Golgi, such as the GFP-tagged temperature sensitive protein tsO45 VSVG from vesicular stomatitis virus in HeLa cells [29], vacuolar hydrolases in *Saccharomyces cerevisiae* [67], components of the cleavage site for cytokinesis in *Drosophila melanogaster* [36], or even virions of the hepatitis C virus in the human hepatoma cell line Huh7.5.1 [68]. Whether these functions in protein trafficking are common or specialized in different cell types is unknown. Moreover, the functions of GOLPH3 in membrane trafficking could be influenced by intrinsic differences in the Golgi apparatus, or by differential mechanisms within the secretory pathway existing in different types of cells. To distinguish between these possibilities, and because there is no specific cargo that we could compare in these breast cell lines, we transiently expressed GFP-tagged proteins that are incorporated in different anterograde and retrograde membrane trafficking pathways at the Golgi: ε-COP-GFP [51], GFP-Rab6A [52], and GFP-GGA1 [45]. We observed similar behaviors in each of the three GFP-tagged proteins in the three cell lines, i.e., the differences were not statistically significant (S4 Fig, S4–S6 Videos), suggesting that the differences in the tubule-vesicular structures containing GFP-GOLPH3 are not the result of major changes in membrane trafficking at the Golgi among these breast cell lines. Together, these results reveal unanticipated differences in the dynamics of membrane transport intermediaries containing GOLPH3. It will be important to determine whether these differences have a functional significance in the trafficking of key protein cargos that contribute to the tumorigenic phenotype.

**Fig 6:**
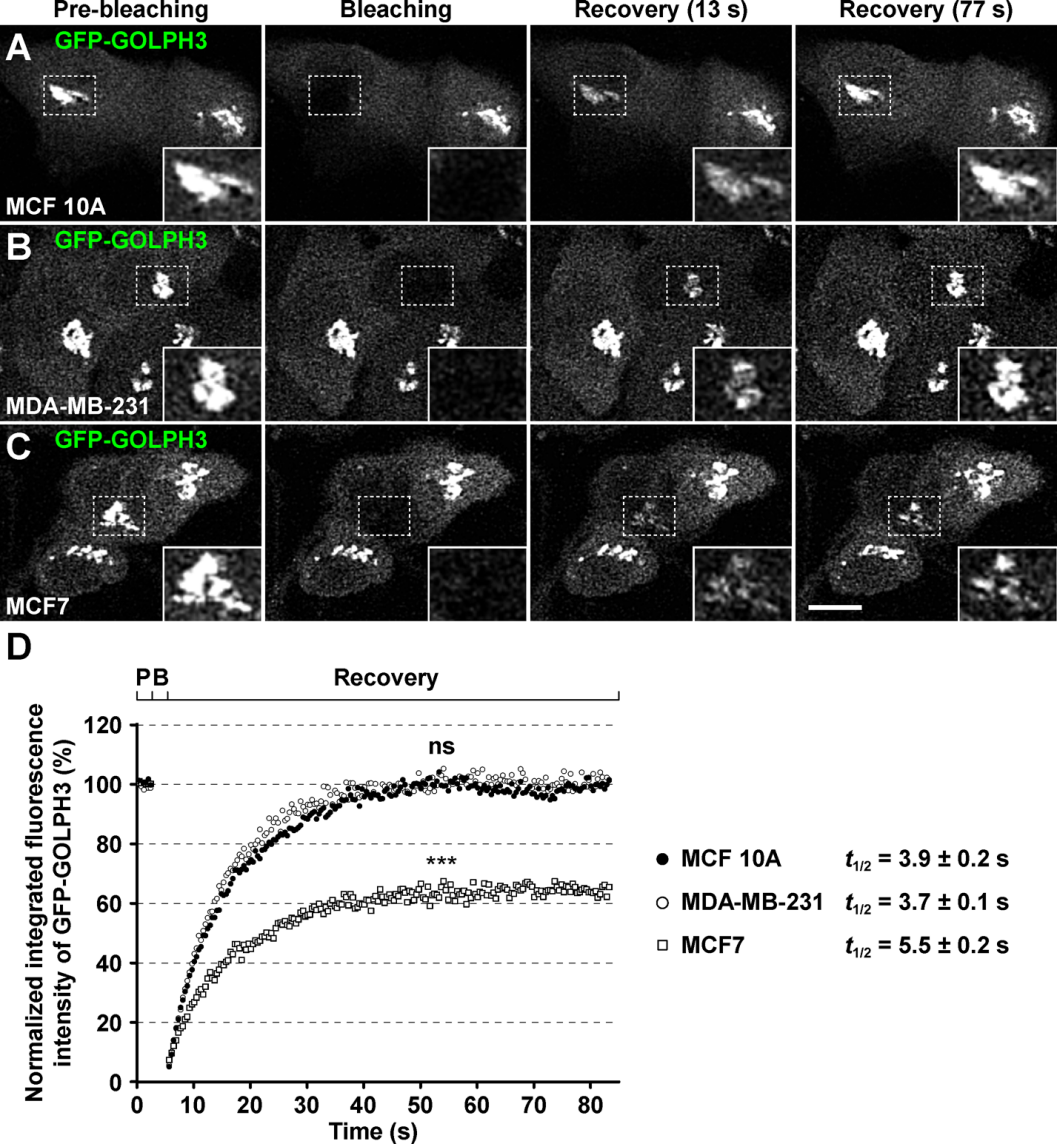
The Golgi-associated fluorescence after photobleaching of GFP-GOLPH3 recovers partially in MCF7 cells. (A-C) MCF 10A (A), MDA-MB-231 (B), and MCF7 (C) cells transiently expressing GFP-GOLPH3 were held in a microscope stage at 37°C. The area indicated by a white dotted-line rectangle in each set of images was bleached with a 488-nm laser set to 100% power. The fluorescence recovery after photobleaching (FRAP) was tracked by laser confocal microscopy with the 488-nm laser set to 2% power. Images were acquired before bleaching (*Pre-bleaching*), immediately after bleaching (*Bleaching*), and during the recovery of the fluorescence (*Recovery*) at approximately every 0.4-sec. Images of a representative experiment performed on each cell line are shown in each set of panels. Two images of the recovery of fluorescence are depicted with the time indicated in parenthesis in seconds. Bar, 10 μm. (D) Plot of the FRAP analysis of GFP-GOLPH3 in MCF 10A (black circles; n = 10), MDA-MB-231 (white circles; n = 10), and MCF7 (white squares; n = 10) cells. *P*, pre-bleaching; *B*; bleaching. For simplicity, error bars are not depicted. *** *P* < 0.001; *ns*, not statistically significant. The halftime (*t*_1/2_) of maximal fluorescence recovery is indicated on the right in seconds (s).

Another dynamic behavior of GFP-GOLPH3 observed in live NRK cells is its rapid exchange between cytosolic and membrane-bound pools, as shown by FRAP analysis [28]. This is a feature shared by several cytosolic proteins that participate in diverse mechanisms important for membrane trafficking at the Golgi, such as the coat protein β-COP or the regulatory GTP binding protein Arf1 [51]. We first evaluated in the three breast cell lines three Golgi proteins of known FRAP parameters: the coat protein GFP-GGA1 [53], the Golgi matrix protein GFP-GRASP55 [55], and the resident transmembrane protein GFP-golgin-84 [54]. We found that the fluorescence recovery of the three proteins was as expected regardless of the cell line, i.e., very slow recovery of GFP-golgin-84, fast recovery of GFP-GRASP55, and faster recovery of GFP-GGA1 (S5 Fig). By similar FRAP analyses, we also found rapid exchange of cytosolic GFP-GOLPH3 in the three breast cell lines, but with important significant differences. While the *t*_1/2_ of maximal recovery in MCF 10A cells (3.9 seconds; Fig 6A and 6D and S5 Fig) and MDA-MB-231 cells (3.7 seconds; Fig 6B and 6D and S5 Fig) were equally fast, in MCF7 cells the *t*_1/2_ was significantly slower (5.5 seconds; Fig 6C and 6D and S5 Fig). The percentage and time of maximal recovery was also different: equally complete (∼100%) at about the same time in MCF 10A cells (26.9 seconds) and MDA-MB-231 cells (27.2 seconds), but partially complete (∼65%) after a longer period of time (48.4 seconds) in MCF7 cells (Fig 6D). Although these results do not definitely demonstrate that endogenous GOLPH3 has correspondingly different behavior in the different cell lines, especially because GFP-GOLPH3 competes with endogenous GOLPH3 for exchange, they suggest that in MCF7 cells the dynamics of membrane association and dissociation is more complex. One possibility is that in MCF7 cells there are two distinct pools of GOLPH3 bound to Golgi membranes, one pool that exchanges rapidly, and a second pool that exchanges much more slowly, by means of a stronger interaction with (a) membrane component(s). One prediction of this hypothesis is that GOLPH3 would be more resistant to extraction from a membrane fraction of MCF7 cells, which is precisely what we found (Fig 2).

### GOLPH3 has different post-translational modifications in different human breast cell lines

**Fig 7:**
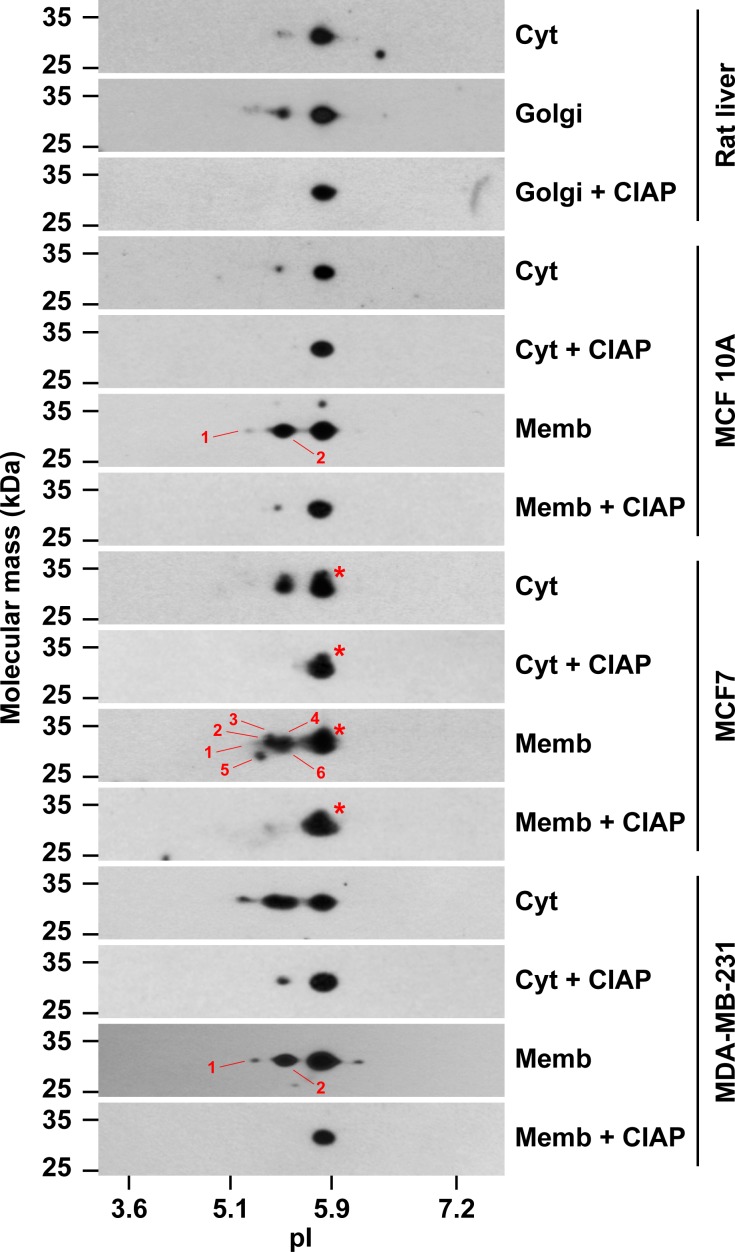
The cytosolic and membrane pools of GOLPH3 are differentially modified in different human breast cell lines. Samples (30 μg of proteins) of rat liver cytosol (*Cyt*), rat liver Golgi membranes, and of cytosolic (*Cyt*) and membrane (*Memb*) fractions from the cell lines indicated at the right were analyzed by two-dimensional gel electrophoresis (2-D GE) and immunoblotting using antibody to GOLPH3. Samples of rat liver Golgi membranes, and of the cytosolic and membrane fractions of each cell line, were dephosphorylated with calf intestine alkaline phosphatase (*CIAP*) before processing for 2-D GE. The position of molecular mass markers is indicated on the left. The position of isoelectric point (*pI*) markers is indicated at the bottom. Red asterisks indicate the position of additional, less abundant, but distinct spots in the samples of MCF7 cells that have slightly slower electrophoretic mobility. Numbers indicate different acidic forms identified in immunoblot films subjected to different exposure times.

Another possible explanation for the FRAP results in MCF7 cells is that cytosolic GOLPH3 also exists in distinct pools that bind with differential kinetics or avidity to Golgi membranes. Such properties could be the result, for instance, of distinct post-translational modification(s). In effect, it has been found by two-dimensional gel electrophoresis (2-D GE) analysis that the rat liver contains forms of GOLPH3 that are differentially phosphorylated, with less phosphorylated forms in the cytosolic pool than in the pool associated to Golgi membranes [27]. This suggests that phosphorylation of GOLPH3 regulates its intracellular localization, which could also influence its function. In fact, some putative phosphorylation sites on GOLPH3, as well as on its orthologue Vps74, have been shown to be functionally important for the DNA-damage-induced Golgi response for cell survival [37], and for hypersensitivity to rapamycin in yeast [69], respectively. Thus, to evaluate whether cytosolic GOLPH3 in the three breast cell lines has unique post-translational modifications, we performed analysis of cytosolic and membrane fractions by 2-D GE, followed by immunoblot with antibody to GOLPH3. We found that, similar to cytosol and Golgi membranes from rat liver, both the cytosol and the membrane fraction of the three cell lines contain a major form of GOLPH3 with an isoelectric point (pI) of ∼5.9 (Fig 7), in agreement with the theoretical pI of ∼6.1 for both rat and human GOLPH3. Also similar to rat liver cytosol, and as expected, we found that the cytosol of MCF 10A cells contains low levels of a more acidic form with a pI of ∼5.4 (Fig 7). In contrast, the membrane fraction of MCF 10A cells contains more abundant amounts of acidic forms of GOLPH3, ranging from pI ∼5.1 to ∼5.4, similar to rat liver Golgi membranes (Fig 7; [27]). Unexpectedly, the cytosol of both MCF7 and MDA-MB-231 cells contained abundant amounts of acidic forms of GOLPH3 (Fig 7). Of note, we detected in the cytosol of MCF7 cells only one additional, but prominent, more acidic form with a pI of ∼5.4, while in the cytosol of MDA-MB-231 cells we also detected other more acidic forms in varied amounts (Fig 7). As expected, the membrane fractions of both MCF7 and MDA-MB-231 cells also contained abundant amounts of more acidic forms of GOLPH3. We noticed, however, some unexpected differences: while the membrane fraction of MCF7 cells contained a higher number of acidic forms than the cytosolic fraction, we observed the opposite for MDA-MB-231 cells, i.e., the membrane fraction contained a lower number of acidic forms than the cytosolic fraction (Fig 7). Interestingly, the patterns of the acidic forms of GOLPH3 in the membrane fractions of MCF 10A and MDA-MB-231 cells were very similar (Fig 7). In contrast, the pattern of acidic forms of GOLPH3 in the membrane fraction of MCF7 cells was more complex (Fig 7). Although the type of modification in all of these more acidic forms of GOLPH3 needs further elucidation, most of them very likely correspond to phosphorylated species, as shown previously by treatment of rat liver Golgi membranes with alkaline phosphatase before 2-D GE analysis [27]. When we performed a similar treatment with alkaline phosphatase, we no longer detected the majority of the acidic forms of both cytosolic and membranes fractions of the three cell lines (Fig 7), suggesting that in fact they corresponded to phosphorylated forms. On the other hand, we also detected forms of GOLPH3 with seemingly slightly higher molecular mass that were resolved exclusively by 2-D GE. These forms, however, were found only in the cytosol and membrane fractions of MCF7 cells, and were resistant to alkaline phosphatase treatment (Fig 7, red asterisks), suggesting that these cells contain additional, or more abundant, or more stable, uncharacterized post-translationally modified GOLPH3 species. Because this complexity of modified forms correlates with membrane association, it is tempting to speculate that phosphorylation accounts for the distinct membrane binding properties of GOLPH3 in MCF7 cells. Another possibility is that a different modification, such as that contained in the forms resistant to alkaline phosphatase, explains the pool of GOLPH3 with different binding properties. Because many Golgi proteins can acquire a number of post-translational modifications other than phosphorylation, including arginine dimethylation [70], or S-nitrosylation, which has also been found in rat GOLPH3 [71], an intriguing further possibility is that differences in post-translational modifications of GOLPH3 among different cancer cell types result in idiosyncratic effects in the tumorigenic phenotypes.

### The cytosol of different human breast cell lines affects distinctly the avidity of GOLPH3 for PtdIns-4-P

**Fig 8:**
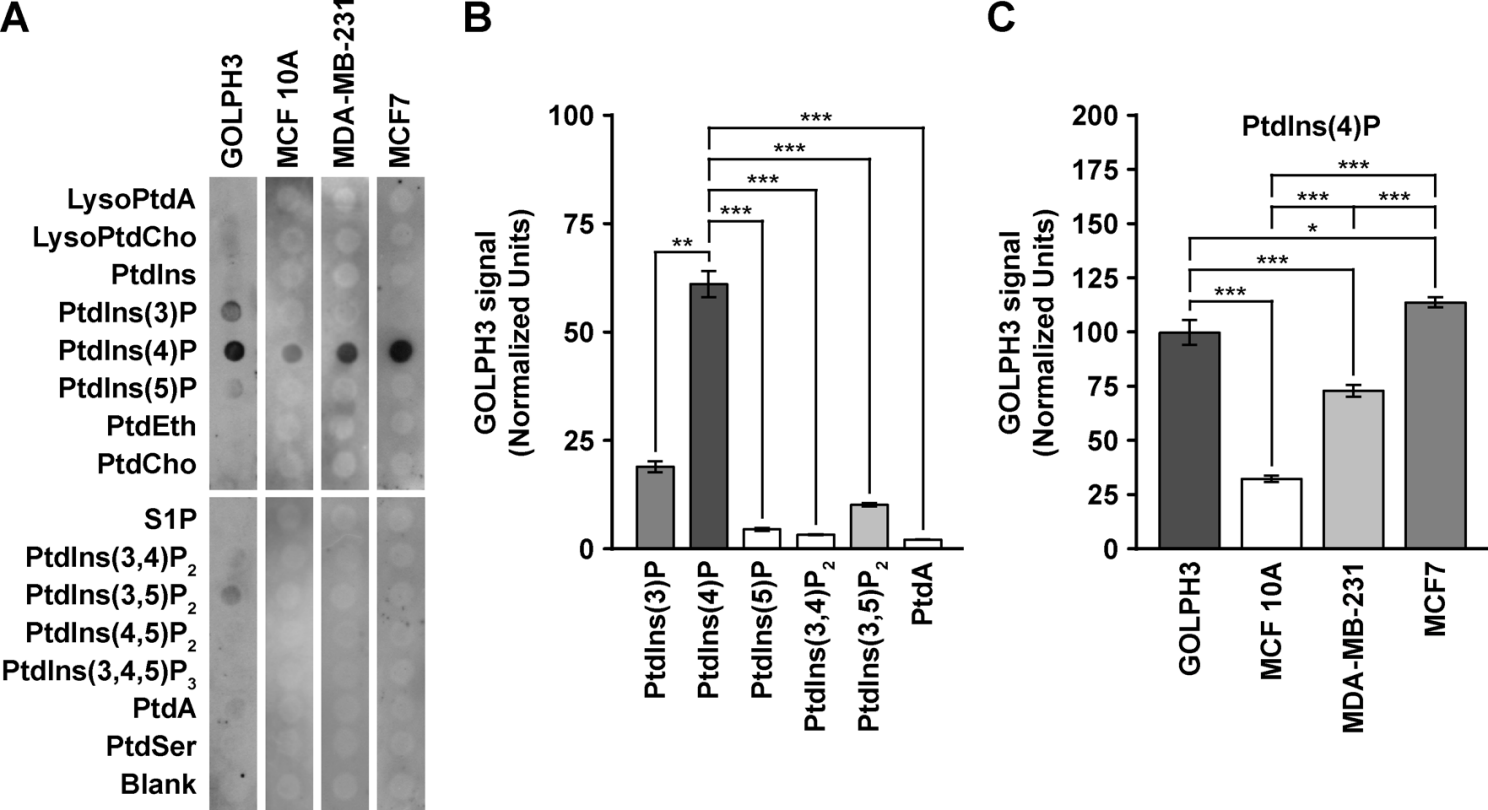
The cytosol of different human breast cell lines affects differently the avidity of GOLPH3 for phosphatidylinositol 4-phosphate. (A) Membranes with the spotted phospholipids indicated on the left were incubated with untreated, recombinant GOLPH3 (*GOLPH3*) or with recombinant GOLPH3 in the presence of cytosolic proteins from the cell lines indicated on the top. Bound recombinant GOLPH3 was detected by immunoblotting with antibody to GOLPH3. *LysoPtdA*, lysophosphatidic acid; *LysoPtdCho*, lysophosphatidylcholine; *PtdIns*, phosphatidylinositol; *PtdIns(3)P*, phosphatidylinositol 3-phosphate; *PtdIns(4)P*, phosphatidylinositol 4-phosphate; *PtdIns(5)P*, phosphatidylinositol 5-phosphate; *PtdEth*, phosphatidylethanolamine; *PtdCho*, phosphatidylcholine; *S1P*, sphingosine 1-phosphate; *PtdIns(3*,*4)P*_*2*_, phosphatidylinositol 3,4-bisphosphate; *PtdIns(3*,*5)P*_*2*_, phosphatidylinositol 3,5-bisphosphate; *PtdIns(4*,*5)P*_*2*_, phosphatidylinositol 4,5-bisphosphate; *PtdIns(3*,*4*,*5)P*_*3*_, phosphatidylinositol 3,4,5-trisphosphate; *PtdA*, phosphatidic acid; *PtdSer*, phosphatidylserine; *Blank*, no lipid. (B) Densitometric quantification of the immunoblot signal of the levels of untreated, recombinant GOLPH3 bound to different phospholipids as shown in (A). (C) Densitometric quantification of the immunoblot signal of the levels of recombinant GOLPH3 bound to phosphatidylinositol 4-phosphate after incubation with cytosolic proteins of the indicated cell lines as shown in (A). * *P* < 0.05; *** *P* < 0.001.

GOLPH3 was also identified in a proteomic lipid-binding screen as a PtdIns-4-P-binding protein [29]. Similarly, in a screen of yeast mutants to identify factors that recruit Vps74, it was found that targeting of both Vps74 and GOLPH3 to yeast Golgi membranes requires the synthesis of PtdIns-4-P by the PtdIns-4-kinase Pik1 [30]. Accordingly, *in vitro*-translated and recombinant (*Escherichia coli*-expressed and purified), Vps74 and human GOLPH3 both bind to PtdIns-4-P [29,30]. Therefore, to assess whether the cytosol of each of the three breast cell lines contains (a) factor(s) that influence(s) the binding of GOLPH3 to PtdIns-4-P, we performed a semi-quantitative lipid-binding assay. For this, we blotted immobilized lipids on a membrane with recombinant, purified human GOLPH3, either left untreated or pre-incubated with corresponding cytosolic fractions, followed by immunoblot analysis with antibody to GOLPH3. We found that our recombinant GOLPH3 binds directly and preferably to PtdIns-4-P, but also in a decreasing, lesser extent to PtdIns-3-P, PtdIns-3,5-P_2_, PtdIns-5-P, and PtdIns-3,4-P_2_ (Fig 8A and 8B). Binding of GOLPH3 to phosphoinositides other than PtdIns-4-P has been previously observed, albeit also to a much lesser extent [29,30]. Remarkably, we also detected binding to phosphatidic acid (Fig 8A and 8B). Although very weak, this is in agreement with the proposed interaction of GOLPH3 and phosphatidic acid for the activation of PI(4)P 5-kinase at the cell surface during cytokinesis in *Drosophila melanogaster* [36]. Strikingly, pre-incubation of GOLPH3 with the cytosolic fraction of either of the breast cell lines resulted in detectable binding to only PtdIns-4-P, precluding the binding to the other phosphoinositides (Fig 8A). Moreover, the avidity for PtdIns-4-P of GOLPH3 pre-incubated with any of the cytosolic fractions was different to that of untreated GOLPH3. While pre-incubation with the cytosolic fraction of MCF7 cells resulted in more binding of GOLPH3 to PtdIns-4-P, significantly less was bound when pre-incubated with the cytosolic fraction of MCF10A or MDA-MB-231 cells (Fig 8A and 8C). We were unable to detect endogenous GOLPH3 bound to any of the phospholipids when we performed the lipid-binding assay with the cytosolic fractions in the absence of recombinant GOLPH3, demonstrating that the amount of recombinant GOLPH3 bound to PtdIns-4-P was not affected by the differential level of endogenous GOLPH3 in the three breast cell lines. On the other hand, immunoblot analysis of recombinant GOLPH3 after the incubation with cytosolic fractions showed an undetectable reduction in its levels (S6 Fig), indicating that the differences in the avidity to phosphoinositides were not a consequence of recombinant GOLPH3 proteolysis. Because the amount of PtdIns-4-P in the Golgi apparatus of MDA-MB-231 cells is more than twice as high as is in MCF7 cells [18], these findings indicate that an uncharacterized cytosolic component of MCF7 cells stimulates the interaction of GOLPH3 with PtdIns-4-P, potentially by means of introducing a post-translational modification such as phosphorylation, or another unidentified modification, as suggested by our 2-D GE analysis (Fig 7). Conversely, the cytosol of both MCF10A and MDA-MB-231 cells seems to have an activity that reduces the binding of GOLPH3 to PtdIns-4-P. Interestingly, although the proportion of GOLPH3 in the membrane fractions of MCF 10A and MDA-MB-231 cells were very similar (∼36%; Fig 1 and S1 Table), pre-treatment of recombinant GOLPH3 with the cytosolic fraction of MDA-MB-231 resulted in ∼2.3 times higher binding to PtdIns-4-P (Fig 8C). This difference could be related to either differential amount of PtdIns-4-P in the Golgi apparatus of MCF 10A and MDA-MB-231 cells, or differential amount of proteins competing for binding to PtdIns-4-P. However, because the amount of recombinant GOLPH3 present in the assay was in such high excess, this last possibility is very unlikely. Alternatively, the cytosol of the three cell lines could contain specific GOLPH3-binding partners that when bound to GOLPH3 either promote or limit its interaction with PtdIns-4-P.

Collectively, the data that we present here allow us to conclude that the cytosol and membrane fractions of these three breast cell lines possesses biochemically distinct pools of GOLPH3, and support the notion that in cancer cells, part of overexpressed GOLPH3 is differentially modified, which could result in varied membrane association properties with diverse gain-of-function events important for oncogenesis. Consistent with this idea, ectopic overexpression of GOLPH3 in MCF7 and MDA-MB-231 cells results in decreased cell-cell adhesion and enhanced cell invasion, which depends on the ability of GOLPH3 to bind to PtdIns-4-P at the Golgi apparatus [18], and seems to proceed by GOLPH3 regulation of the sialylation of *N*-glycans on integrins [34]. Similarly, ectopic overexpression of GOLPH3 in MCF7 and MDA-MB-231 cells results in increased cell proliferation, which correlates with down-regulation of the cyclin-dependent kinase (CDK) inhibitors p21^Cip1^, p27^Kip1^, and p57^Kip2^, and up-regulation of the cyclin D1, by a mechanism that seems to involve suppression of the transcription factor FOXO1 [9]. Still, the sequence of events that connect GOLPH3 with these phenotypes, and how they are triggered by GOLPH3, remain unknown. Moreover, another apparently dissimilar function elicited by ectopic overexpression of GOLPH3 in MDA-MB-231 cells is the promotion of mitochondrial biogenesis, which enhances anabolic tumor growth [39]. Furthermore, cumulative evidence indicates that GOLPH3 participates in several other cellular processes critical for other cancer cells, emerging as an attractive therapeutic target [26]. However, in order to consider GOLPH3 in new anti-cancer strategies, it will be important to characterize its multiple cellular functions in different types of cancer cells.

## Supporting Information

S1 Fig The redistribution of GOLPH3 upon BFA treatment is distinct in different human breast cell lines. (A) Cultures of the cells indicated on the top were left untreated (-) or treated (+) with 5 μg/ml BFA for 60 min. Membrane (*M*) and cytosolic (*C*) fractions were prepared, and equivalent amounts of each fraction (15 μg of membrane proteins and 5 μg of cytosolic proteins) were subjected to SDS-PAGE followed by immunoblotting using antibodies to the proteins indicated on the right. The position of molecular mass markers is indicated on the left. (B) Densitometric quantification of the immunoblot signal of the levels of GOLPH3 in membrane (*M*) or cytosolic (*C*) fractions as shown in (A). Bar represents the mean ± standard deviation of the amount of immunoblot signal normalized with the signal for β-actin. Note that to detect the redistribution of proteins from membranes to cytosol upon BFA treatment the gels were loaded with proteins of membrane and cytosolic fractions in a ratio 3:1, instead of 1:1 used in the immunoblots shown in Fig 1.
(TIF)

S2 Fig GFP-GOLPH3 localizes at the Golgi apparatus in breast cell lines. (A-C) MCF 10A (A), MDA-MB-231 (B), and MCF7 (C) cells transiently expressing GFP-GOLPH3 were fixed, permeabilized, and immunolabeled with rabbit polyclonal antibody to GOLPH3, and mouse monoclonal antibody to GM130. Secondary antibodies were Alexa-594-conjugated donkey anti-rabbit IgG (red channel), and Alexa-647-conjugated donkey anti-mouse IgG (blue channel). Stained cells were examined by fluorescence microscopy. Merging red, green, and blue channels generated the fourth image on each row; yellow indicates overlapping localization of the red and green channels, cyan indicates overlapping localization of the green and blue channels, magenta indicates overlapping localization of the red and blue channels, and white indicates overlapping localization of the three channels. Insets show 2.5x magnifications. Note the lower level of endogenous GOLPH3 in untransfected MCF10A cells (arrows in A) compared to that of MDA-MB-231 and MCF7 cells. Bar, 10 μm.
(TIF)

S3 Fig GFP-GOLPH3 distributes in cytosolic and membrane fractions in a similar manner as endogenous GOLPH3 in the breast cell lines MCF 10A, MCF7, and MDA-MB-231. (A-C) Cell homogenates from the indicated cell lines that were either left untreated (*-*) or transfected to transiently express GFP-GOLPH3 (*+*) were used to prepare cytosolic (*C*) and membrane (*M*) fractions. Equivalent amounts of each fraction (10 μg of proteins) were subjected to SDS-PAGE and immunoblotting using antibodies to the proteins indicated on the right, or to GFP to detect GFP-GOLPH3. The position of molecular mass markers is indicated on the left. (D-F) Densitometric quantification of the immunoblot signal of the levels of GOLPH3 and GFP-GOLPH3 in cytosolic (*C*) and membrane (*M*) fractions as shown in (A-C). Bar represents the mean ± standard deviation of the amount of immunoblot signal normalized with the signal for β-actin, and also for the total amount of protein in each fraction. * *P* < 0.05; *** *P* < 0.001; *ns*, not statistically significant.
(TIF)

S4 Fig The dynamic behavior of ε-COP-GFP, GFP-Rab6A, and GFP-GGA1 is similar in the breast cell lines MCF 10A, MCF7, and MDA-MB-231. (A, D, G) MCF 10A, MDA-MB-231, and MCF7 cells transiently expressing ε-COP-GFP (A), GFP-Rab6A (D), or GFP-GGA1 (G) were held in a microscope stage at 37°C and examined by fluorescence microscopy. The time after initiation of imaging is shown in the bottom right corner of each panel in minutes:seconds. Images are representative of 15–20 videos of up to 200 seconds of recording. Filled, yellow arrows indicate tubule-vesicular structures moving centripetally. Filled, red arrows indicate tubule-vesicular structures moving centrifugally. Empty arrows indicate the initial position of mobile structures. Bars, 2 μm. (B-C, E-F, H-I) The number of tubule-vesicular structures moving centrifugally (B, E and H), or the number of tubule-vesicular structures moving centripetally (C, F, and I), were quantified from videos corresponding to 60 seconds (ε-COP-GFP) or 20 seconds (GFP-Rab6A and GFP-GGA1) of imaging. Bar represents the mean ± standard deviation of the observed profiles (n = 6); *10A*: MCF 10A; *231*: MDA-MB-231; *7*: MCF7; *ns*, not statistically significant.
(TIF)

S5 Fig The Golgi-associated fluorescence recovery after photobleaching of GFP-GRASP55, GFP-golgin-84, and GFP-GGA1 is similar in different breast cell lines. MCF 10A (A), MDA-MB-231 (B), and MCF7 (C) cells, transiently expressing either of the indicated GFP-tagged proteins, were held in a microscope stage at 37°C. The fluorescence of equivalent areas of the Golgi was bleached with a 488-nm laser set to 100% power. The fluorescence recovery after photobleaching (FRAP) was tracked by laser confocal microscopy with a 488-nm laser set to 2% power. Images were acquired before bleaching (*P*), immediately after bleaching (*B*), and during the recovery of the fluorescence (*Recovery*) at approximately every 0.4-sec. The average of the normalized integrated fluorescence intensity was plotted over time for each GFP-tagged protein. For comparison, the FRAP analysis of GFP-GOLPH3 (Fig 6) is also shown. GFP-GOLPH3: white circles (n = 10); GFP-GGA1: red circles (n = 4); GFP-GRASP55: blue squares (n = 5); and GFP-golgin-84: green rhombuses (n = 3). For simplicity, error bars are not depicted. The halftime (*t*_1/2_) of maximal fluorescence recovery is indicated on the right in seconds (s). The *t*_1/2_ values of each GFP-tagged protein between different cell lines were not statistically significant.
(TIF)

S6 Fig The levels of recombinant GOLPH3 are not reduced by incubation with cytosolic fractions. Aliquots of the incubations mixtures used in the lipid-binding assay (see ‘Materials and Methods‘ and Fig 8) were analyzed to evaluate proteolysis of recombinant GOLPH3. Aliquots representing 1/1000^th^ of the incubation mixtures without (-) or with (+) an aliquot of a cytosolic fraction of the indicated cell lines, before (-) or after (+) the incubation condition indicated on the right, were processed by SDS-PAGE and immunoblotting using antibodies to the proteins indicated on the right. The position of molecular mass markers is indicated on the left.
(TIF)

S1 Table Amount of proteins in cytosol and membrane fractions, and relative amount of GOLPH3 in the corresponding fractions of MCF 10A, MCF7 and MDA-MB-231 cells. (DOCX)

S1 Video Behavior of GFP-GOLPH3 in live MCF 10A cells. (MOV)

S2 Video Behavior of GFP-GOLPH3 in live MDA-MB-231 cells. (MOV)

S3 Video Behavior of GFP-GOLPH3 in live MCF7 cells. (MOV)

S4 Video Behavior of ε-COP-GFP in live breast cell lines cells. (MOV)

S5 Video Behavior of GFP-Rab6A in live breast cell lines cells. (MOV)

S6 Video Behavior of GFP-GGA1 in live breast cell lines cells. (MOV)
