# Supplementary figures and images for: Distinct Biochemical Pools of Golgi Phosphoprotein 3 in the Human Breast Cancer Cell Lines MCF7 and MDA-MB-231
Source: PLoS One. 2016 Apr 28;11(4):e0154719. doi: 10.1371/journal.pone.0154719 (PMC4849736; doi:10.1371/journal.pone.0154719)

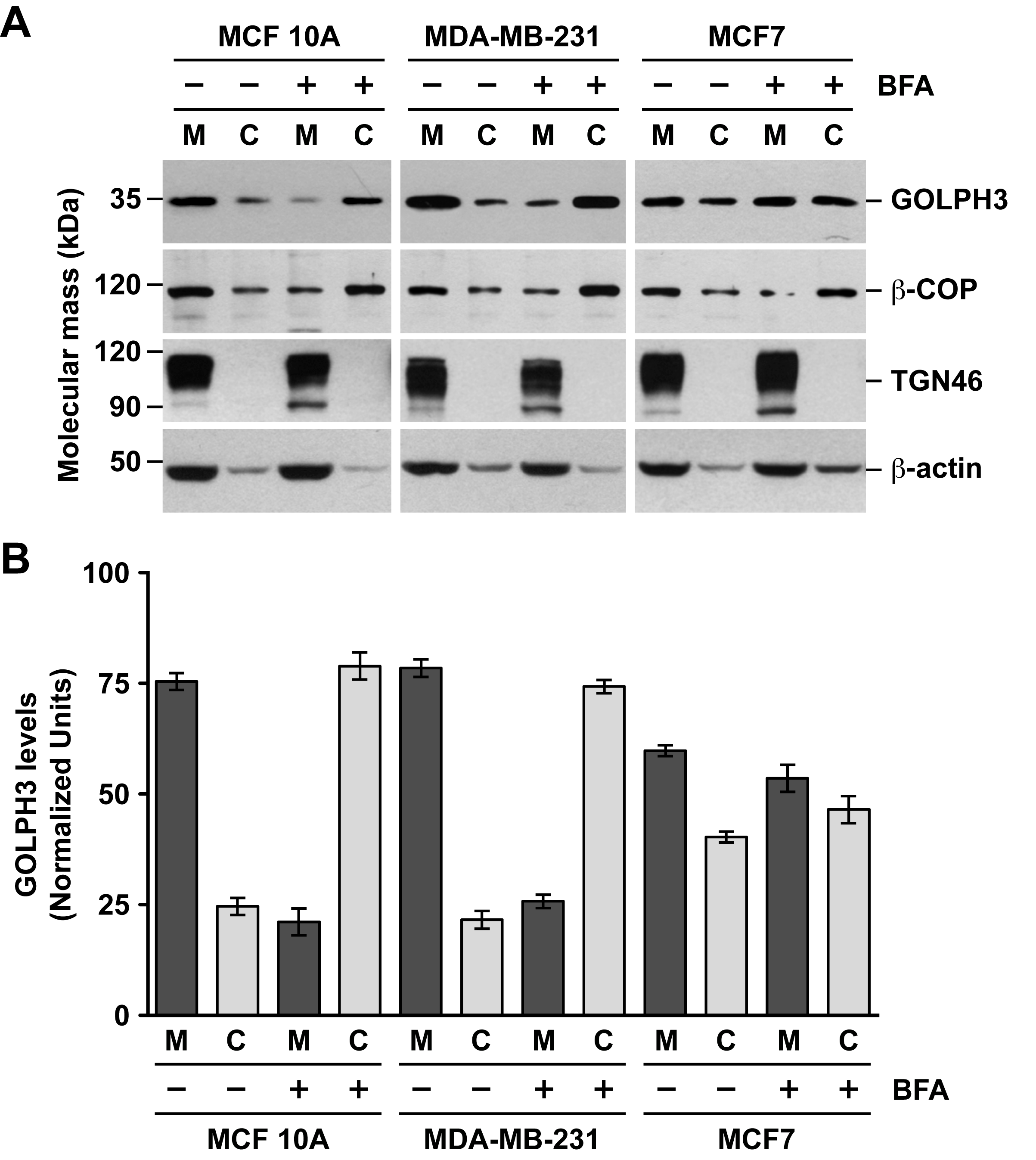

Supplement: S1 Fig — (A) Cultures of the cells indicated on the top were left untreated (-) or treated (+) with 5 μg/ml BFA for 60 min. Membrane (M) and cytosolic (C) fractions were prepared, and equivalent amounts of each fraction (15 μg of membrane proteins and 5 μg of cytosolic proteins) were subjected to SDS-PAGE followed by immunoblotting using antibodies to the proteins indicated on the right. The position of molecular mass markers is indicated on the left. (B) Densitometric quantification of the immunoblot signal of the levels of GOLPH3 in membrane (M) or cytosolic (C) fractions as shown in (A). Bar represents the mean ± standard deviation of the amount of immunoblot signal normalized with the signal for β-actin. Note that to detect the redistribution of proteins from membranes to cytosol upon BFA treatment the gels were loaded with proteins of membrane and cytosolic fractions in a ratio 3:1, instead of 1:1 used in the immunoblots shown in Fig 1. (TIF) [file pone.0154719.s001.tif]

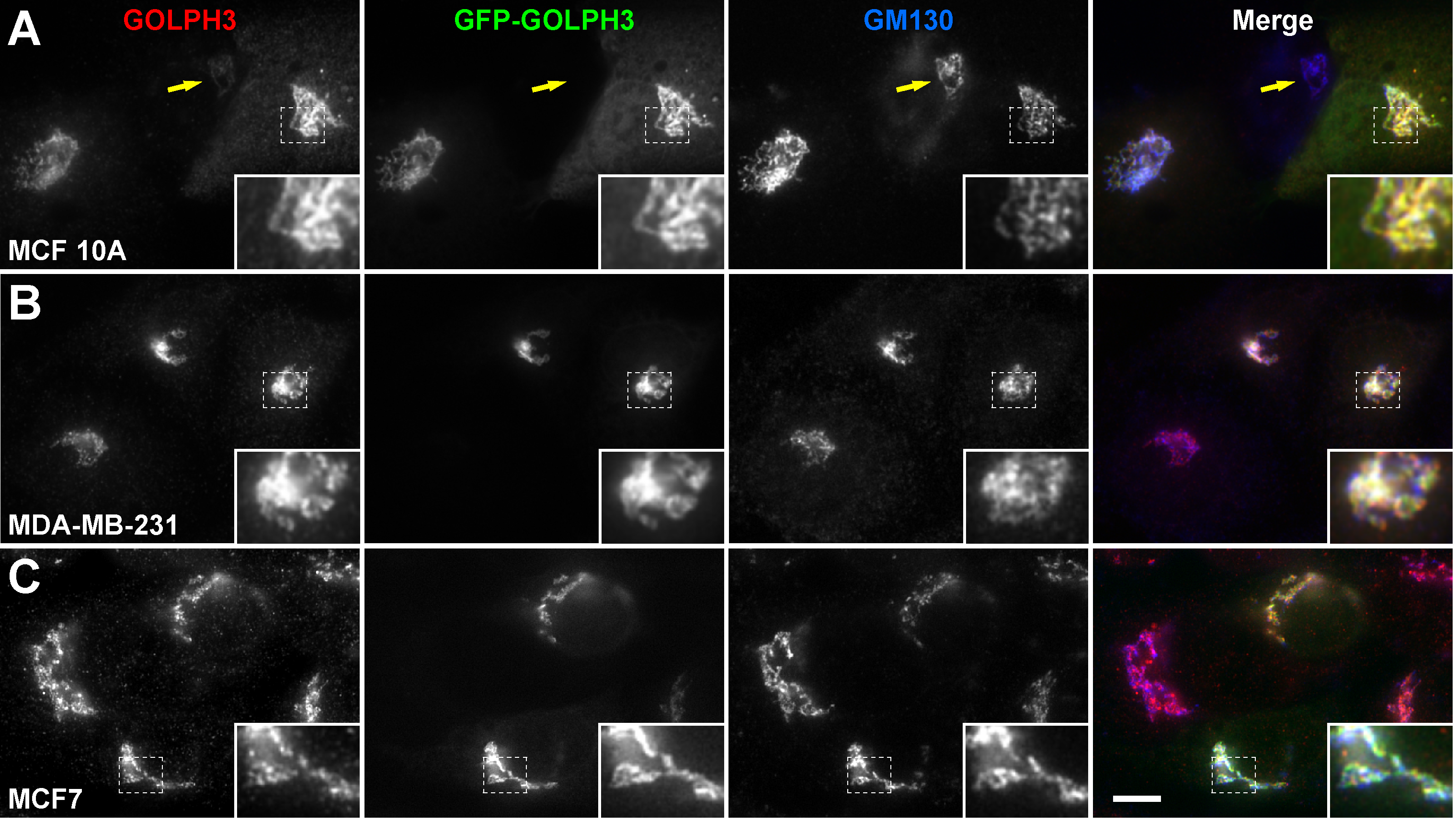

Supplement: S2 Fig — (A-C) MCF 10A (A), MDA-MB-231 (B), and MCF7 (C) cells transiently expressing GFP-GOLPH3 were fixed, permeabilized, and immunolabeled with rabbit polyclonal antibody to GOLPH3, and mouse monoclonal antibody to GM130. Secondary antibodies were Alexa-594-conjugated donkey anti-rabbit IgG (red channel), and Alexa-647-conjugated donkey anti-mouse IgG (blue channel). Stained cells were examined by fluorescence microscopy. Merging red, green, and blue channels generated the fourth image on each row; yellow indicates overlapping localization of the red and green channels, cyan indicates overlapping localization of the green and blue channels, magenta indicates overlapping localization of the red and blue channels, and white indicates overlapping localization of the three channels. Insets show 2.5x magnifications. Note the lower level of endogenous GOLPH3 in untransfected MCF10A cells (arrows in A) compared to that of MDA-MB-231 and MCF7 cells. Bar, 10 μm. (TIF) [file pone.0154719.s002.tif]

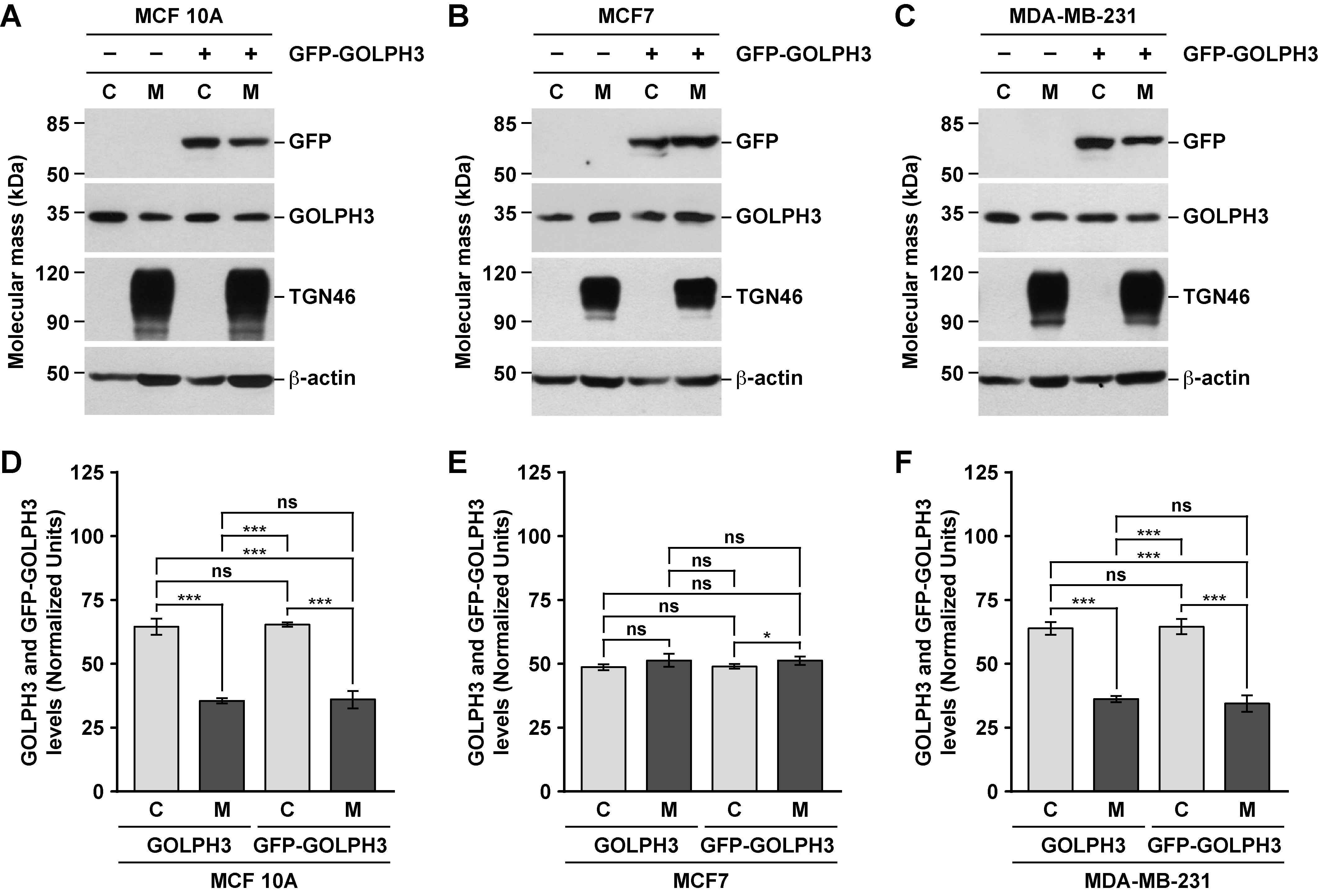

Supplement: S3 Fig — (A-C) Cell homogenates from the indicated cell lines that were either left untreated (-) or transfected to transiently express GFP-GOLPH3 (+) were used to prepare cytosolic (C) and membrane (M) fractions. Equivalent amounts of each fraction (10 μg of proteins) were subjected to SDS-PAGE and immunoblotting using antibodies to the proteins indicated on the right, or to GFP to detect GFP-GOLPH3. The position of molecular mass markers is indicated on the left. (D-F) Densitometric quantification of the immunoblot signal of the levels of GOLPH3 and GFP-GOLPH3 in cytosolic (C) and membrane (M) fractions as shown in (A-C). Bar represents the mean ± standard deviation of the amount of immunoblot signal normalized with the signal for β-actin, and also for the total amount of protein in each fraction. * P < 0.05; *** P < 0.001; ns, not statistically significant. (TIF) [file pone.0154719.s003.tif]

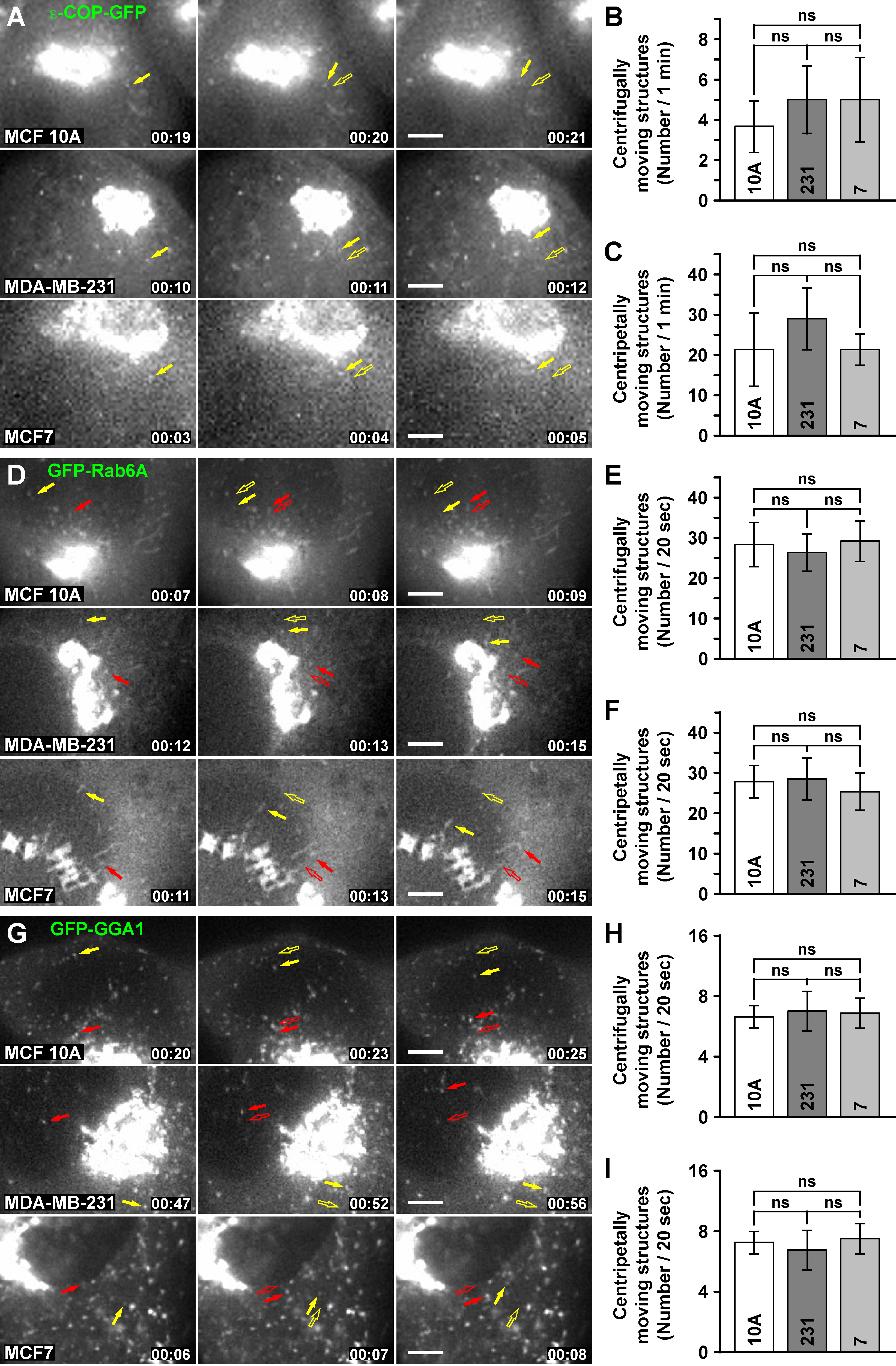

Supplement: S4 Fig — (A, D, G) MCF 10A, MDA-MB-231, and MCF7 cells transiently expressing ε-COP-GFP (A), GFP-Rab6A (D), or GFP-GGA1 (G) were held in a microscope stage at 37°C and examined by fluorescence microscopy. The time after initiation of imaging is shown in the bottom right corner of each panel in minutes:seconds. Images are representative of 15–20 videos of up to 200 seconds of recording. Filled, yellow arrows indicate tubule-vesicular structures moving centripetally. Filled, red arrows indicate tubule-vesicular structures moving centrifugally. Empty arrows indicate the initial position of mobile structures. Bars, 2 μm. (B-C, E-F, H-I) The number of tubule-vesicular structures moving centrifugally (B, E and H), or the number of tubule-vesicular structures moving centripetally (C, F, and I), were quantified from videos corresponding to 60 seconds (ε-COP-GFP) or 20 seconds (GFP-Rab6A and GFP-GGA1) of imaging. Bar represents the mean ± standard deviation of the observed profiles (n = 6); 10A: MCF 10A; 231: MDA-MB-231; 7: MCF7; ns, not statistically significant. (TIF) [file pone.0154719.s004.tif]

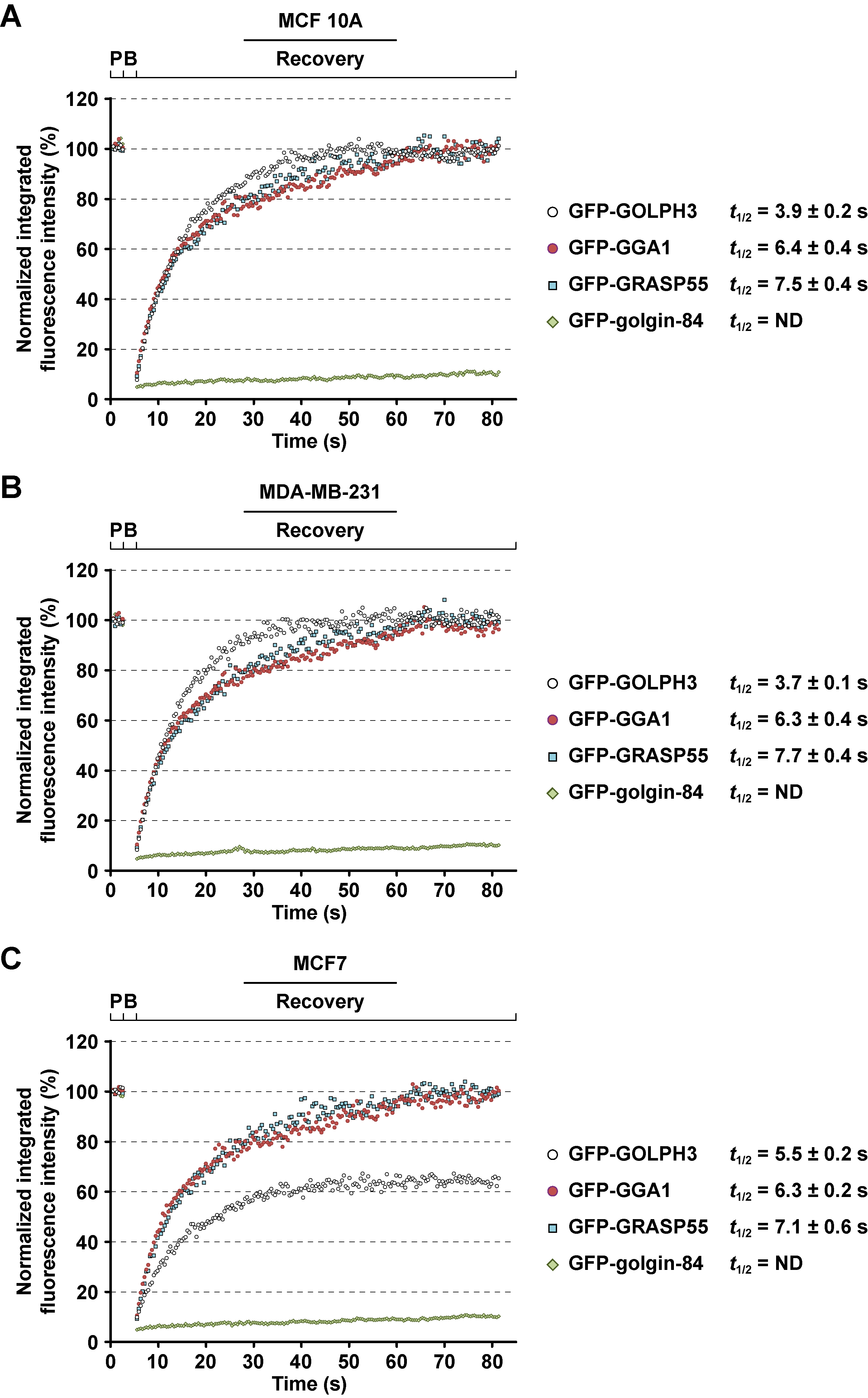

Supplement: S5 Fig — MCF 10A (A), MDA-MB-231 (B), and MCF7 (C) cells, transiently expressing either of the indicated GFP-tagged proteins, were held in a microscope stage at 37°C. The fluorescence of equivalent areas of the Golgi was bleached with a 488-nm laser set to 100% power. The fluorescence recovery after photobleaching (FRAP) was tracked by laser confocal microscopy with a 488-nm laser set to 2% power. Images were acquired before bleaching (P), immediately after bleaching (B), and during the recovery of the fluorescence (Recovery) at approximately every 0.4-sec. The average of the normalized integrated fluorescence intensity was plotted over time for each GFP-tagged protein. For comparison, the FRAP analysis of GFP-GOLPH3 (Fig 6) is also shown. GFP-GOLPH3: white circles (n = 10); GFP-GGA1: red circles (n = 4); GFP-GRASP55: blue squares (n = 5); and GFP-golgin-84: green rhombuses (n = 3). For simplicity, error bars are not depicted. The halftime (t1/2) of maximal fluorescence recovery is indicated on the right in seconds (s). The t1/2 values of each GFP-tagged protein between different cell lines were not statistically significant. (TIF) [file pone.0154719.s005.tif]

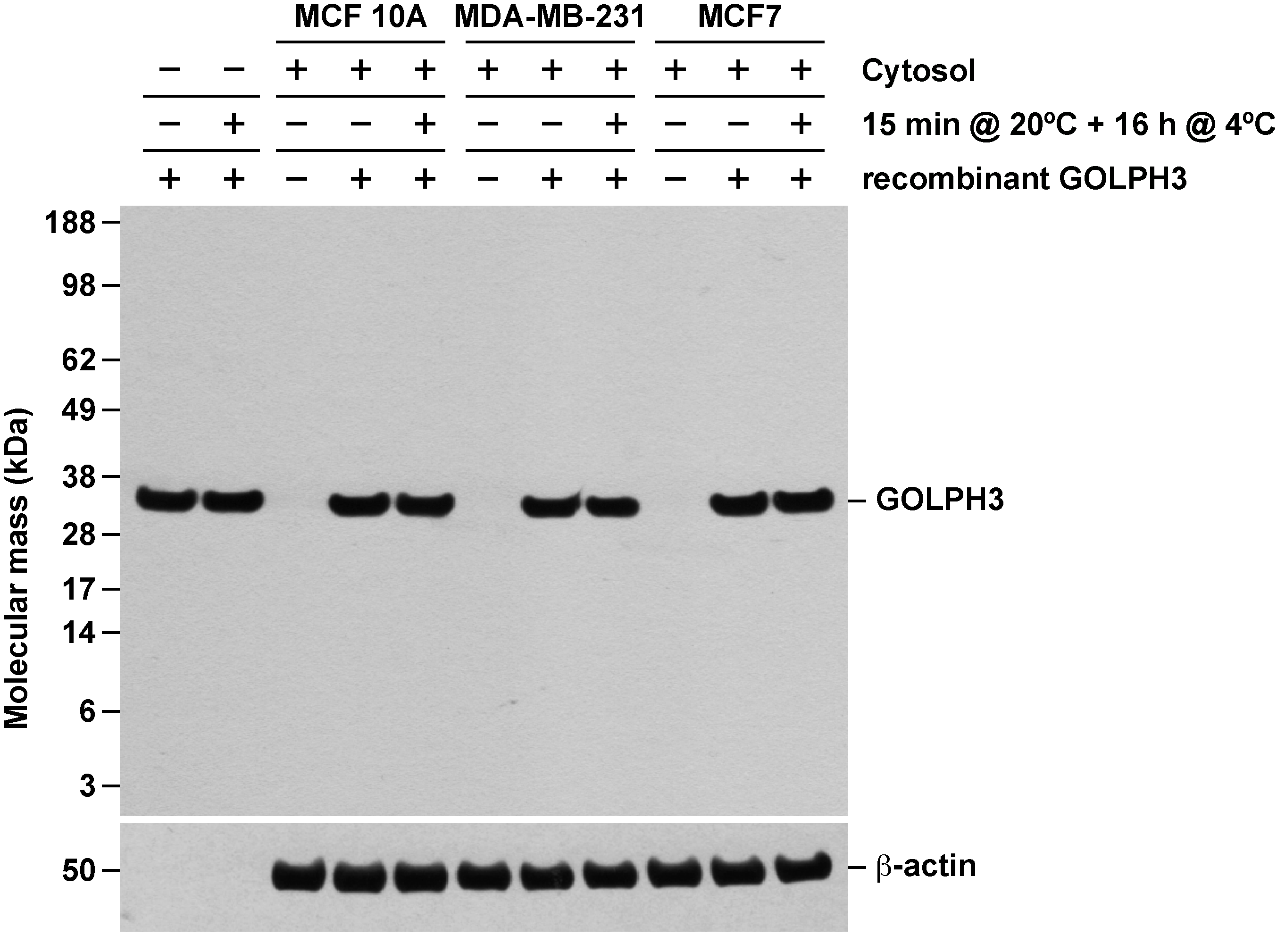

Supplement: S6 Fig — Aliquots of the incubations mixtures used in the lipid-binding assay (see ‘Materials and Methods‘ and Fig 8) were analyzed to evaluate proteolysis of recombinant GOLPH3. Aliquots representing 1/1000th of the incubation mixtures without (-) or with (+) an aliquot of a cytosolic fraction of the indicated cell lines, before (-) or after (+) the incubation condition indicated on the right, were processed by SDS-PAGE and immunoblotting using antibodies to the proteins indicated on the right. The position of molecular mass markers is indicated on the left. (TIF) [file pone.0154719.s006.tif]
