# Supplementary material for: Distinct Biochemical Pools of Golgi Phosphoprotein 3 in the Human Breast Cancer Cell Lines MCF7 and MDA-MB-231
Source: PLoS One. 2016 Apr 28;11(4):e0154719. doi: 10.1371/journal.pone.0154719 (PMC4849736; doi:10.1371/journal.pone.0154719)
Supplement: S1 Table — (DOCX) [file pone.0154719.s007.docx]

**S1 Table. Amount of proteins in cytosol and membrane fractions, and relative amount of GOLPH3 in the corresponding fractions of MCF 10A, MCF7 and MDA-MB-231 cells**

| Cell line | Protein (μg)^a^ | | Protein (%)^b^ | | GOLPH3 densitometric signal (arbitrary units)^c^ | | GOLPH3 normalized signal (%)^d^ | |
| --- | --- | --- | --- | --- | --- | --- | --- | --- |
|  | *Cytosol* | *Membrane* | *Cytosol* | *Membrane* | *Cytosol* | *Membrane* | *Cytosol* | *Membrane* |
| MCF 10A | 646.8 ± 11.6 | 400.5 ± 18.2 | 61.7 ± 1.0 | 38.3 ± 1.0 | 9749 ± 477 | 8633 ± 259 | 64.5 ± 3.2 | 35.5 ± 1.1 |
| MCF7 | 722.4 ± 7.5 | 486.7 ± 6.1 | 59.7 ± 0.2 | 40.3 ± 0.2 | 9429 ± 227 | 14748 ± 732 | 48.7 ± 1.2 | 51.3 ± 2.5 |
| MDA-MB-231 | 1619.0 ± 26.7 | 1015.9 ± 13.5 | 61.4 ± 0.6 | 38.6 ± 0.6 | 14576 ± 571 | 13140 ± 431 | 63.8 ± 2.5 | 36.2 ± 1.2 |

^a^ Average (n=3) ± SD of the content of protein in the cytosol (700 μl) and membrane (300 μl) fractions recovered after subcellular fractionation. For more explanation see ‘Subcellular Fractionation, Membrane Fractions Stripping and Size Exclusion Chromatography’ in ‘Experimental Procedures’.

^b^ Average (n=3) ± SD of the percentage of protein content in each fraction relative to the total protein recovered (cytosol + membrane).

^c^ Average (n=5) ± SD of the amount of GOLPH3 immunoblot signal from gels loaded with equal amount of protein (10 μg) from each fraction.

^d^ Average (n=5) of the amount of GOLPH3 immunoblot signal (^c^) normalized relative to the amount of protein in each fraction (^b^), expressed as % ± SD (depicted in Fig. 1C).
